# Long-term risks of adverse kidney outcomes after acute kidney injury: a systematic review and meta-analysis

**DOI:** 10.1093/ndt/gfaf093

**Published:** 2025-05-27

**Authors:** Denise M J Veltkamp, Cindy P Porras, Christina M Gant, Wouter M Tiel Groenestege, Maarten B Kok, Marianne C Verhaar, Wouter W van Solinge, Saskia Haitjema, Robin W M Vernooij

**Affiliations:** Department of Nephrology and Hypertension, University Medical Center Utrecht, Utrecht University, Utrecht, the Netherlands; Central Diagnostic Laboratory, University Medical Center Utrecht, Utrecht University, Utrecht, The Netherlands; Department of Nephrology and Hypertension, University Medical Center Utrecht, Utrecht University, Utrecht, the Netherlands; Julius Center for Health Sciences and Primary Care, University Medical Center Utrecht, Utrecht University, Utrecht, the Netherlands; Department of Nephrology and Hypertension, University Medical Center Utrecht, Utrecht University, Utrecht, the Netherlands; Central Diagnostic Laboratory, University Medical Center Utrecht, Utrecht University, Utrecht, The Netherlands; Saltro BV, Unilabs Netherlands, Utrecht, the Netherlands; Department of Nephrology and Hypertension, University Medical Center Utrecht, Utrecht University, Utrecht, the Netherlands; Central Diagnostic Laboratory, University Medical Center Utrecht, Utrecht University, Utrecht, The Netherlands; Central Diagnostic Laboratory, University Medical Center Utrecht, Utrecht University, Utrecht, The Netherlands; Department of Nephrology and Hypertension, University Medical Center Utrecht, Utrecht University, Utrecht, the Netherlands; Central Diagnostic Laboratory, University Medical Center Utrecht, Utrecht University, Utrecht, The Netherlands; Julius Center for Health Sciences and Primary Care, University Medical Center Utrecht, Utrecht University, Utrecht, the Netherlands

**Keywords:** acute kidney injury, chronic kidney disease, kidney failure, long-term outcomes, major adverse kidney event

## Abstract

**Background:**

Acute kidney injury (AKI) is associated with increased risks of incidence or progression of chronic kidney disease (CKD), kidney failure (KF), or major adverse kidney events (MAKE), however, it remains unclear which individuals have higher risks. Hence, we systematically reviewed the literature to explore differences in kidney dysfunction risks between AKI stages, AKI durations, and clinical settings.

**Methods:**

We performed a systematic search in PubMed and Embase to identify studies that examined at least one outcome of interest in individuals with AKI versus without AKI, with a minimum follow-up of one year. Hazard/odds ratios (HR/OR) were pooled using random effects models. Heterogeneity across patient and disease characteristics was examined using subgroup and meta-regression analyses.

**Results:**

We searched 70 studies, encompassing 1 838 668 individuals, including 165 715 with AKI. All studies were of moderate to high quality. Individuals with AKI had higher risks of CKD incidence [AKI 25.8%/no AKI 8.7%; HR 2.36 [95% confidence interval (CI) 1.77–2.94)]], CKD progression [AKI 43.1%/no AKI 35.6%; HR 1.83 (95%CI 1.26–2.40)], KF [AKI 2.9%/no AKI 0.5%; HR 2.64 (95%CI 2.03–3.25)], and MAKE [AKI 59.0%/no AKI 32.7%; OR 2.77 (95%CI 2.01–3.53)]. The pooled effect estimates for CKD incidence after AKI lasting <3 days remained significant [OR 2.37 (95%CI 1.68–3.07)], even in individuals with AKI stage 1 only [HR 1.49 (95%CI 1.44–1.55)]. Diabetes mellitus, hypertension, requiring acute dialysis, cardiovascular surgery, or coronary artery disease were associated with higher CKD incidence or progression risks.

**Conclusions:**

Risks for kidney dysfunction were higher for all individuals with AKI. Risk estimates were heterogeneous between patient subgroups, based on AKI stage, AKI duration, and clinical setting, yet even individuals with the lowest stage or shortest duration of AKI remained at higher risk for CKD incidence or progression. This highlights the need to develop tailored follow-up strategies to recognize kidney function decline post-AKI and initiate kidney protective measures in a timely fashion.

## Graphical Abstract

**Graphical Abstract:**
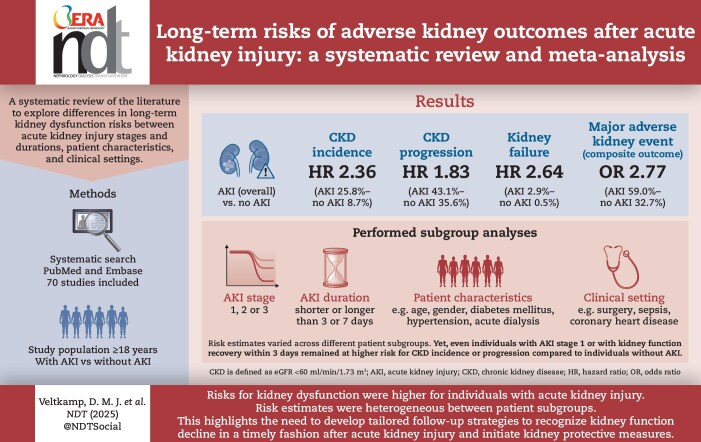

KEY LEARNING POINTS **What was known:**

- Acute kidney injury (AKI) is associated with increased risks of adverse kidney outcomes, and incidence or progression of chronic kidney disease (CKD), kidney failure (KF), or major adverse kidney events (MAKE).
- Timely identification of CKD is imperative for initiating preventive measures that may impede further cardiorenal consequences.

**This study adds:**

- The risks of CKD incidence or progression, as well as KF, were higher among individuals with more severe AKI stages or longer AKI durations. Notably, even individuals with the lowest AKI stage or a short AKI duration of <3 days had increased risks of these outcomes.
- Diabetes mellitus, hypertension, and acute kidney replacement therapy were associated with increased risk of CKD incidence/progression after AKI.
- Risks of kidney dysfunction after AKI were different between clinical settings; the highest risks in comparison to individuals without AKI were seen in the context of cardiovascular surgery.

**Potential impact:**

- This review raises awareness of the association of AKI and CKD incidence, CKD progression, KF, and MAKE. Even individuals with the lowest AKI stage or short-lasting AKI showed increased risk for CKD incidence or progression. It emphasizes the importance of vigilance for post-AKI decline in kidney function.
- Risks for adverse kidney outcomes were heterogeneous between patient subgroups, based on AKI stage, AKI duration, and clinical setting. This highlights the need to develop tailored strategies to promptly recognize kidney function decline post-AKI and initiate kidney protective measures.

## INTRODUCTION

Acute kidney injury (AKI), an abrupt worsening of the kidney function, occurs in ∼10%–20% of hospitalized individuals [1, 2]. The etiology of AKI is highly heterogeneous, and it frequently emerges secondary to underlying conditions. The association of AKI with

higher risk of incidence or progression of chronic kidney disease (CKD) and increased risk of kidney failure (KF) has been established [3, 4, 5, 6]. CKD and KF are significant and growing public health challenges worldwide, especially because of their association with an increased risk of cardiovascular morbidity, the need for kidney replacement therapy, and premature mortality [5, 7, 8]. In addition, CKD and KF are associated with an increased overall symptom burden and reduced health-related quality of life [9]. Timely identification of CKD is imperative for initiating preventive measures that may impede further cardiorenal consequences, including blood pressure and glycemic control, use of renin–angiotensin–aldosterone system inhibitors and statins, and nephrologist follow-up, which are shown to be effective among AKI survivors with CKD [10, 11, 12].

A comprehensive overview of individualized long-term risks among individuals with AKI is absent. In particular, the significance of AKI duration in relation to AKI-associated adverse kidney outcomes, with respect to CKD incidence, CKD progression, and KF, has not been thoroughly explored systematically. The most recent systematic review on long-term outcomes after AKI, conducted by See *et al.* showed an association between AKI and long-term mortality [5]. However, the analyses on the association of AKI with CKD and KF were hampered by a small number of studies, and, notably, the outcome CKD was not divided according to CKD incidence in individuals without pre-existing CKD, and CKD progression in individuals with pre-existing CKD. Additionally, the importance of AKI duration on long-term kidney dysfunction remains largely unclear. A review by Coca *et al.* reported on the association of AKI duration with survival [13]. Another review by Mehta *et al.* on post-AKI outcomes stratified by AKI duration could only include two studies on CKD incidence [14]. In our review we aimed to explore whether AKI is associated with increased risks of CKD incidence, progression of CKD, KF, and major adverse kidney events (MAKE), stratified per AKI stage, AKI duration, and clinical setting [5, 13, 14, 15]. Understanding the differential risks with respect to the long-term adverse kidney outcomes post-AKI might contribute to the improvement of tailored follow-up protocols for individuals with AKI. This may ultimately reduce the burden of AKI-associated adverse kidney outcomes.

## MATERIALS AND METHODS

### Protocol and search strategy

This systematic review and meta-analysis was registered in PROSPERO (CRD42023425871) and reported according to the Preferred Reporting Items for Systematic Reviews and Meta-analyses (PRISMA) guideline and Meta-analysis of Observational Studies in Epidemiology guidelines [16, 17, 18]. We performed a search in PubMed and Embase from 2018 to 2 October 2024. The search strategy included among others the terms AKI, CKD, KF, and MAKE (Table S1). See *et al.* performed a comparable review in 2018 (and included studies from January 2004 until February 2018) [5]. We included the studies from before 2018 that were selected by See *et al.* in our full-text screening.

### Exposure and outcomes

The exposure of interest in the study was AKI, and its definition was in accordance with the criteria established by Kidney Disease: Improving Global Outcomes (KDIGO), Acute Kidney Injury Network (AKIN), or Risk, Injury, Failure, Loss, End-stage kidney disease (RIFLE, introduced by the Acute Dialysis Quality Initiative (ADQI)), as reported by the original studies [19, 20, 21]. RIFLE stages Risk, Injury, and Failure were analyzed as KDIGO and AKIN stages 1, 2, and 3, respectively. The outcomes of interest were CKD incidence, CKD progression, KF, and MAKE. MAKE is a composite endpoint of death, initiation of kidney replacement therapy, or a decrease in kidney function. CKD incidence was analyzed in individuals without prior CKD, and CKD progression in individuals with pre-existing CKD (“acute on chronic kidney injury”). Individuals were identified as having CKD from CKD stage 3A (or eGFR <60 ml/min/1.73 m^2^) and beyond, following the KDIGO criteria.

### Screening and data extraction

Title and abstract screening were performed by R.W.M.V. and D.M.J.V., and the full-text assessment by C.P.P., R.W.M.V., and D.M.J.V. Disagreements were resolved by discussion. Studies were considered eligible if (i) the study was an observational cohort study; (ii) at least one of the outcomes of interest (CKD incidence, CKD progression, KF, or MAKE) was reported; (iii) the minimum age was 18 years; (iv) a control group was included containing individuals without AKI; or (v) the mean follow-up time was at least one year post-AKI. Studies were excluded if (i) they were case-control studies, clinical trials to prevent the influence of intervention from affecting our analyses, case-reports, commentaries, study protocols, or conference abstracts; (ii) the clinical setting in which the AKI occurred was restricted to pregnancy, chronic liver disease, chronic heart failure, transplantation (solid organs or stem-cells), nephrectomy, or COVID-19; (iii) the study was not written in English, or (iv) the manuscript could not be retrieved.

### Data extraction and risk of bias assessment

Data were extracted by D.M.J.V. and R.W.M.V. The risk of bias assessment was performed by D.M.J.V. and C.P.P. using the Newcastle-Ottawa Scale [22]. Herewith, the selection of the study groups (e.g. representativeness of the cohort with individuals in clinical practice), the comparability of the groups (e.g. the groups were equal concerning age, gender, baseline kidney function, the percentage of diabetes mellitus and hypertension, or confounding adjustments made for these variables), and the ascertainment of the outcome (e.g. adequate follow-up length or percentage of individuals loss to follow-up) were assessed using a “star” system. Any study could obtain maximum four, two, and three stars per category, respectively. Studies with a total of seven or more stars were considered as high-quality studies, with five or six stars as moderate-quality studies, and below five stars as low-quality studies. Publication bias was assessed using Egger’s test for funnel plot asymmetry and Egger's linear regression test [23].

### Statistical analyses

Meta-analyses were performed for each outcome (CKD incidence, CKD progression, KF, and MAKE) using a random effects model to calculate the weighted means. Since the studies represent different populations, in different healthcare settings, clinical heterogeneity was expected, and a random effect model was deemed more appropriate than the fixed-effects model. Outcomes reported as hazard ratios (HR) or odds ratios (OR) were both included in the analyses. If the event rate of the outcome was reported, the OR was calculated following the *Cochrane Handbook for Systematic Reviews of Interventions* [24]. Studies involving the same cohort were included only once.

Using meta-analyses, the overall associations of AKI, compared with non-AKI, with CKD incidence or progression (combined), KF, and MAKE were examined. In subgroup analyses the outcomes were (i) stratified for individuals without pre-existing CKD (CKD incidence) and with pre-existing CKD (CKD progression) separately, (ii) studied per AKI stage, and (iii) per AKI duration. Also (iv), the combination of AKI stage and AKI duration was studied. AKI duration was divided in two groups: shorter than 3 days versus 3 days and longer, and shorter than 7 days versus 7 days and longer. These durations were based on regularly used cut-off durations in existing literature: shorter than 3 days (“transient AKI”), 3 to 7 days (“persistent AKI”), or 7 days to 3 months (“acute kidney disease”) [25, 26, 27, 28]. In all meta-analyses, heterogeneity was assessed with the *I*^2^ statistic.

Additionally, meta-regression analyses were performed to analyze effect modification on the association between AKI and the outcomes if at least 10 studies could be included. The analyzed variables included mean age, the percentage of male individuals, mean baseline eGFR (estimated glomerular filtration rate), the proportion of individuals having diabetes mellitus or hypertension or that required acute dialysis, follow-up duration, and study size. Preferably, HRs were included in the meta-regression; however, if HRs were not reported ORs were used instead.

Finally, the outcome prevalences and relative risks were calculated for each clinical setting. Event rates were aggregated per clinical setting if the event rates and baseline characteristics, including age, gender, and kidney function, were extractable. The studies were pragmatically classified into categories: cardiovascular surgery non-cardiovascular surgery, out of hospital cardiac arrest (OHCA), coronary artery disease (including patients with myocardial infarction, or patients who underwent coronary angiography/percutaneous coronary intervention), infection or sepsis, neurovascular event, hematologic disease, intrinsic renal disease (e.g. lupus nephritis or IgA-nephropathy), or post-renal obstruction. The remaining studies were categorized as “hospital.” In practice, the cause of AKI will mostly be multifactorial. However, these categories might help to relate the results of this review to clinical practice.

A *P* value <.05 was considered significant. Analyses were performed using R version 4.3.2 and RStudio v.2023.09.1+494 [29, 30].

### Sensitivity analysis

In sensitivity analyses, we analyzed the overall association of AKI with the outcomes in (i) studies that reported the results as HRs, (ii) studies that defined AKI following the KDIGO or AKIN criteria, and (iii) studies that adjusted for baseline kidney function (or studies with a difference in baseline eGFR of <10% between individuals with AKI and without AKI).

## RESULTS

**Figure 1:**
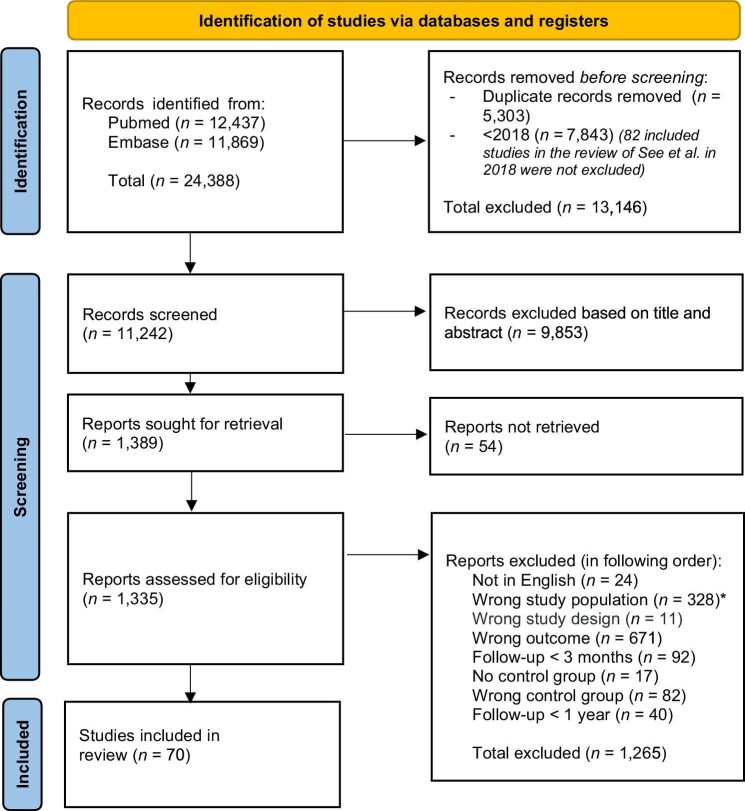
Prisma flow diagram. *Including the reports that were excluded based on clinical settings: pregnancy, solid organ transplantation, stem-cell transplantation, chronic liver disease or heart failure, nephrectomy, and COVID-19 infection.

We identified 11 242 studies, of which 1389 were assessed based on full text. In total, 70 studies were included in the review, which consisted of 66 different cohorts (Fig. 1). Studies of the same cohort are counted as one. In total, 40 reported on CKD incidence and/or CKD progression (29 reported on CKD incidence, and 9 on CKD progression separately), 35 on KF, and 7 on MAKE. Reasons for exclusion in the full-text analysis are reported in Table S2.

Overall, 165 715 individuals with AKI and 1 672 953 individuals without AKI were included. The median sample size was 475 [IQR 133–1936] individuals with AKI, and 1357 [IQR 442–8403] individuals without AKI. The mean age was 64.7 and 61.5 years, and the mean proportion of women across studies was 34% and 38% for individuals with and without AKI, respectively. In total, 28 studies were performed in post-surgery setting (17 cardiovascular surgery, 11 non-cardiovascular surgery). Further, one study was performed in OHCA survivors, one in hematologic disease patients, eight in the context of coronary artery disease, two in the context of intrinsic renal disease, and five in the context of an infection or sepsis. The 22 other studies were labeled as hospital (in one study the outcomes were stratified in two clinical settings) [31]. In total, 17 studies were performed in Europe, 24 in North-America, 1 in South-America, 2 in Oceania, and 22 in Asia. Of all the studies, 13 were conducted prospectively (Fig. S1).

### Exposure and outcome definitions

Most studies defined AKI by the KDIGO criteria, 12 studies used the AKIN criteria, and 5 followed the RIFLE definition. The outcome definition of CKD progression was not consistent throughout all nine included studies; three studies used a minimum of 50% of eGFR decrease, four studies reported CKD progression as a worsening of at least one CKD stage compared to the initial CKD stage, one as 35% eGFR decrease, and one as new eGFR <30 ml/min/1.73 m^2^. The outcome definitions of the included studies that reported on CKD incidence, KF, or MAKE, were more homogeneous. Definitions are reported in the meta-analyses plots.

### Risk of bias

Risk of bias analysis showed that most studies were of moderate to high quality. Some studies did not adjust for age, gender, baseline kidney function, diabetes mellitus, or hypertension, leading to the risk of reduced comparability. Many studies did not report on loss to follow-up (Table S3). Egger's linear regression test and the funnel plots showed a significant funnel plot asymmetry for CKD incidence (*P* < .05) in studies that reported HRs, which might indicate publication bias (Fig. S2).

### CKD incidence or progression

**Figure 2:**
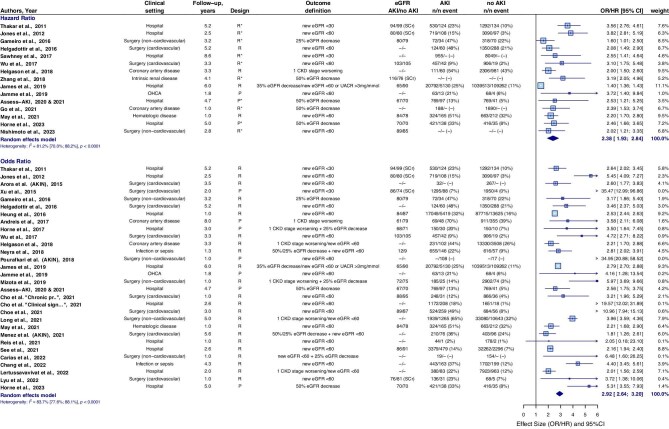
CKD incidence or progression in individuals with AKI compared to individuals without AKI. The combined outcome of CKD incidence or CKD progression occurred in 27.4% (14 365/52 380) of individuals with AKI and in 11.5% (142 110/1 234 776) of individuals without AKI. The weighted mean HR for CKD incidence and CKD progression of James *et al.* 2019 was included. *Outcome is adjusted for baseline kidney function (eGFR). CI, confidence interval; P, prospective; R, retrospective; SCr, serum creatinine; UACR, urinary albumin-to-creatinine ratio.

**Figure 3:**
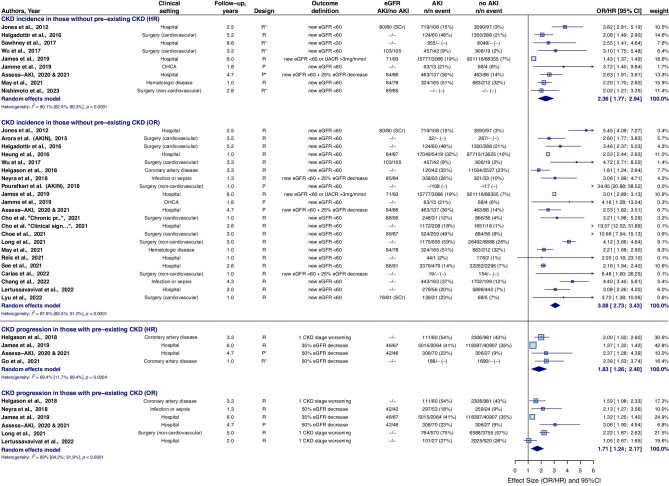
Subanalysis on CKD incidence and CKD progression separately in individuals with AKI compared to individuals without AKI. CKD incidence occurred in 25.8% (11 068/42 854) of individuals with AKI and in 8.7% (95 168/1 096 517) of individuals without AKI. CKD progression in individuals with pre-existing CKD occurred in 43.1% (2 844/6 594) of individuals with AKI and in 35.6% (46 214/129 881) of individuals without AKI. UACR, urinary albumin-to-creatinine ratio. *Outcome is adjusted for baseline kidney function (eGFR). CI, confidence interval; P, prospective; R, retrospective; SCr, serum creatinine; UACR, urinary albumin-to-creatinine ratio.

CKD incidence or progression occurred in 27.4% of individuals with AKI and in 11.5% of individuals without AKI. Individuals with AKI had a pooled HR of 2.38 (95%CI 1.93–2.84) and pooled OR of 2.92 (95%CI 2.64–3.20) for development of CKD or CKD progression, compared to individuals without AKI (Fig. 2). Separately, the pooled HR was 2.36 (95%CI 1.77–2.94) for CKD incidence and 1.83 (95%CI 1.26–2.40) for CKD progression (Fig. 3).

**Figure 4:**
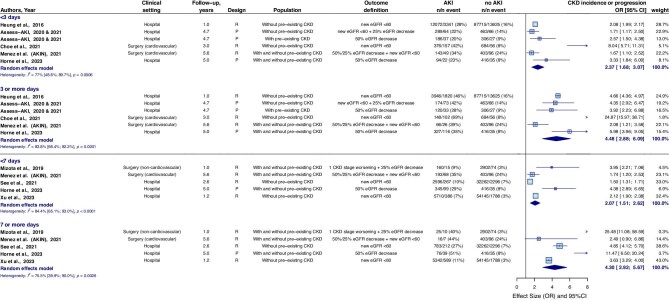
CKD incidence or CKD progression in individuals with AKI compared to individuals without AKI, stratified for AKI duration. Only ORs included in this meta-analysis. The Assess-AKI studies, See *et al.* and Xu *et al.* also reported HRs. Subanalysis only including HRs showed pooled HRs of 2.24 (95%CI 0.87–3.61), 4.54 (95%CI 1.83–7.25), 2.49 (95%CI 1.44–3.54), and 3.91 (95%CI 2.69–5.41) for AKI duration of <3, 3 or more, <7, and 7 or more days, respectively. CI, confidence interval; P, prospective; R, retrospective; SCr, serum creatinine.

The higher the AKI stage, the higher the risk for CKD incidence or CKD progression. AKI stage 1 showed a pooled HR/OR (combined) of 2.40 (95%CI 1.88–2.92) compared to individuals without AKI (Fig. S3a–c). The pooled HR/OR (combined) was 2.62 (95%CI 1.71–3.53) in individuals with a recovered kidney function within 3 days post-AKI, and 2.64 (95%CI 1.84–3.45) with a recovered kidney function within 7 days post-AKI, compared to individuals without AKI (Fig. 4). Studies of the ASSESS-AKI cohort reported there was still an increased risk in individuals with AKI that lasted <1 day for CKD incidence [HR 2.23 (95%CI 1.46–3.42)], although not for CKD progression [HR 0.94 (95%CI 0.38–23.0)] [32].

The combination of AKI stage and duration was analyzed in two studies for CKD incidence in individuals without pre-existing CKD [33, 34]. Individuals with AKI stage 1 during < 3 days and 3 days or longer had pooled HRs of 1.49 (95%CI 1.44–1.55) and 2.96 (95%CI 2.13–3.80), compared to individuals without AKI, respectively (the analysis was mainly weighted by the study of Heung *et al.*) [33].

Individuals with AKI in de context of coronary artery disease had the highest prevalence of CKD incidence or CKD progression [35]. However, individuals with AKI after cardiovascular surgery showed the highest relative risk of CKD incidence or progression versus individuals without AKI [34, 36–38, 39] (Table 2).

**Table 1:** Study characteristics.

							AKI				AKI/no AKI					
Authors	Year	Country	Design	Setting	AKI definition	FU (y)^a^	AKI (*n*)	Stage 1/2/3/2 or 3 (%)	Acute KRT (%)	No AKI (*n*)	Age (y)	Male (%)	BL kidney function^b^	BL CKD ≥ 3A (%)	DM (%)	HT (%)
Choi *et al.* [55]	2010	US	R	Hospital	AKIN	5.7	3 060	80/–/–/20	11	14 265	46/44	99/98		14/6	11/7	25/19
James *et al.* [56]	2011	Canada	R	Coronary artery disease	AKIN	1.6	1 420	77/–/–/23		13 362	68/63	70/72	65/75	46/22	35/25	72/65
Thakar *et al.* [57]	2011	US	R	Hospital	KDIGO	5.2	530	88/–/–/12		1 292			94/99 (SCr)			
Wu *et al.* [58]	2011	Taiwan	P	Hospital	RIFLE	4.8	4 158	58/26/18/42	4	4 727	61/57	60/60	69/76 (SCr)	0/0	23/16	38/35
Jones *et al.*[59]	2012	US	R	Hospital	KDIGO	2.5	719	31/36/33/69		3 090	64/59	54/47	80/80 (SCr)	0/0	42/21	75/45
Bucaloiu *et al.* [60]	2012	US	R	Hospital	KDIGO	3.3	1 610	71/23/7/29		3 652	63/63	47/45	97/97	0/0	29/27	58/56
Chawla *et al.* [61]	2014	US	R	Coronary artery disease	KDIGO	1.4	9 633	78/14/7/22		18 921	70/66	99/98	70/76	0/0	45/34	61/58
Rydén *et al.* [62]	2014	Sweden	R	Surgery (cardiovascular)	AKIN	4.3	3 721	85/–/–/15		25 609	70/66	80/78	69/78	38/17	31/22	68/55
Arora *et al.*[63]	2015	US	R	Surgery (cardiovascular)	AKIN	3.5	86	78/8/2/10		381						
Xu *et al.* [36]	2015	China	P	Surgery (cardiovascular)	KDIGO	2.0	1 295	66/18/16/34	4	1 950	56/51	74/59	86/74 (SCr)	0/0	11/8	31/24
Gameiro *et al.* [64]	2016	Portugal	R	Surgery (non-cardiovascular)	KDIGO	3.2	72	79/17/4/21	3	318	70/60	61/48	80/79		22/19	60/49
Helgadottir *et al.* [65]	2016	Iceland	R	Surgery (cardiovascular)	KDIGO	5.2	184	66/15/20/34	10	1 526	69/66	79/82	72/83	33/12	25/15	69/64
Heung *et al.* [33]	2016	US	R	Hospital	KDIGO	1.0	17 049	91/3/5/9		8 7715	63/62	96/95	84/87	0/0	46/34	86/75
Grams *et al.* [66]; Grams *et al.* [67]	2016	US	R	Surgery (non-cardiovascular)	KDIGO	3.8	19 025	76/15/9/24	2	142 160	64/64		80/80			
Andreis *et al.* [35]	2017	Italy	P	Coronary artery disease	KDIGO	8.0	69			911	72/66	71/72	61/79	55/16	32/19	80/75
Chawla *et al.* [68]	2017	US	R	Infection or sepsis	KDIGO	1.8	13 390			33 067	70/68	98/97	74/78		32/24	54/48
Chew *et al.* [69]	2017	Singapore	P	Surgery (cardiovascular)	AKIN	4.4	777	78/–/–/22	6	1 889	62/58	78/81	72/85	17/5	53/42	84/71
Horne *et al.* [70]	2017	UK	P	Hospital	KDIGO	3.0	150	70/16/14/30	1	150	72/71	59/57	68/71	34/31	20/15	
Palomba *et al.* [71]	2017	Brazil	P	Surgery (cardiovascular)	KDIGO	1.0	88			127				0/0		
Sawhney *et al.* [72]	2017	Canada	R	Hospital	KDIGO	8.6	1 966	69/21/10/31		12 685	73/68	49/42	75/66	51/36	13/5	
Wu *et al.* [39]	2017	China	R	Surgery (cardiovascular)	KDIGO	3.3	457	74/16/10/26		906	55/51	59/46	103/105	0/0	13/11	38/27
Azzalini *et al.* [73]	2018	Italy	R	Coronary artery disease	AKIN	2.0	86		0	809				28/17		
Helgason *et al.* [74]	2018	Iceland	R	Coronary artery disease	KDIGO	3.3	231	76/10/15/29	8	13 330	70/65	69/70		48/17	27/14	69/66
Neyra *et al.* [75]	2018	US	R	Infection or sepsis	KDIGO	1.3	1 497	49/–/–/51		1 135	66/66	55/51	66/64	45/47	23/21	
Pourafkari *et al.* [76]	2018	US	P	Surgery (non-cardiovascular)	AKIN	1.0	1 272	73/11/16/27		6 292	70/65	96/98	115/100 (SCr)	25/25	30/20	45/41
Takahashi *et al.* [77]	2018	US	R	Surgery (non-cardiovascular)	AKIN	10.0	10		0	58	73/74	80/48	18/25	100/95	50/41	90/97
Zhang *et al.* [78]^E^	2018	China	R	Intrinsic renal disease	KDIGO	4.1	82			41	36/33	58/41	116/76 (SCr)	42/14		67/30
Chaudhury *et al.* [79]	2019	US	R	Hospital	KDIGO adjusted^c^	1.8	150			450				100/100		
James *et al.* [80]	2019	Canada	R	Hospital	KDIGO	6.0	20 792			1 039 513	65/52	50/43	65/90	24/11	6/3	10/7
Jamme *et al.* [81]	2019	France	P	OHCA	KDIGO	1.8	65	6/8/33/41	40	68				0/0		
Mizota *et al.* [82]	2019	Japan	R	Surgery (non-cardiovascular)	KDIGO	1.0	258	83/12/5/17		3493	66/66	82/60	72/75		27/16	53/29
Wang *et al.* [83]	2019	US	R	Surgery (cardiovascular)	RIFLE	1.3	24		17	96				46/24	4/21	92/88
Zhang *et al.* [84]	2019	Australia	R	Hospital	KDIGO	2.8	2 199		4	4 166	68/64	57/52		74/69		
Yeh *et al.* [85]	2019	Taiwan	R	Hospital	RIFLE	1.0	1 905			4 141	69/67	53/59	20/31	96/88	54/39	68/60
Foxwell *et al.* [86]	2020	UK	R	Hospital	KDIGO	1.0	548	69/20/11/31		571	70/55	55/46	81/102	28/8	28/10	53/30
Jiang *et al.* [87]	2020	Taiwan	R	Coronary artery disease	AKIN	2.9	54			1 046	69/68	56/75	42/52		65/52	78/73
Lysak *et al.* [88]	2020	US	R	Surgery (non-cardiovascular)	KDIGO	5.0	7 817			8 357	74/73	54/48		19/6	22/20	60/61
Wang *et al.* [89]	2020	China	R	Surgery (cardiovascular)	AKIN	4.2	382	72/20/8/28	6	550						
ASSESS-AKI Cohort [40] [90] [32] [91]	2020, 2021	US & Canada	P	Hospital	KDIGO	4.7	769	74/16/11/26	3	769	64/65	68/58	67/70	40/40	51/35	
Cho *et al.* [37]	2021	Korea	R	Surgery (cardiovascular)	KDIGO	1.0	284		2	906	66/61	54/52	88/95	0/0	22/15	48/44
Cho *et al.* [92]	2021	Korea	R	Hospital	KDIGO	2.6	1 419	95/–/–/5		1 783	65/63	75/63		15/7	18/11	
Choe *et al.* [34]	2021	Korea	R	Surgery (cardiovascular)	KDIGO	3.0	652	81/–/–/19		1357	64/64	72/74	89/87	0/0	26/25	55/48
Glasbey [93]	2021	UK	P	Surgery (non-cardiovascular)	KDIGO	1.0	475	68/–/–/32		3 029	67/62	63/54		22/13	20/14	
Go *et al.* [94 ]^E^	2021	US	R	Coronary artery disease	KDIGO	1.0	188			1 690				100/100		
Long *et al.* [95]	2021	Iceland	R	Surgery (non-cardiovascular)	KDIGO	5.0	3 516	33/–/–/67		43 817	74/68	59/54	95/79 (SCr)	44/22	12/6	27/17
May *et al.* [96]	2021	US	R	Hematologic disease	KDIGO	1.0	355	65/19/17/35		714	67/66	68/54	84/78	9/7	21/16	36/34
Menez *et al.* [97]	2021	US	R	Surgery (cardiovascular)	AKIN	5.6	210	89–/–/92		403						
Reis *et al.* [98]	2021	Portugal	R	Hospital	KDIGO	1.0	87			315				49/43		
See *et al.* [42]	2021	Australia	R	Hospital	KDIGO	2.6	4 827			57 665	62/54	52/50	86/91	0/0	23/11	23/5
Carias *et al.*[99]	2022	Portugal	R	Surgery (non-cardiovascular)	KDIGO	1.0	23			159	60/58	65/69	81/93	17/3	26/12	44/36
Chang *et al.* [100]	2022	Taiwan	R	Infection or sepsis	KDIGO	4.3	443		17	1 702	52/48	79/67		0/0	19/17	37/30
Chen, L. *et al.* [101]	2022	China	P	Coronary artery disease	AKIN	1.0	41			361	69/59	76/88	80/91	17/8	22/17	58/47
Lertussavavivat *et al.* [102]	2022	Thailand	R	Hospital	KDIGO	2.0	578			8 448	66/64	48/45		31/31	34/30	52/51
Lyu *et al.* [38]	2022	China	R	Surgery (cardiovascular)	KDIGO	1.0	187		53	97	54/52	70/73	76/81 (SCr)	0/0	9/6	70/57
Nishio *et al.* [103]	2022	Japan	R	Surgery (cardiovascular)	KDIGO	5.0	115	56/–/–/44	17	99	71/74	72/54	62/63		15/14	83/77
Privratsky *et al.* [104]	2022	Iceland	R	Surgery (non-cardiovascular)	KDIGO	1.0	490	79/12/9/21		7 009				53/17		
Chou *et al.* [105]	2023	Taiwan	R	Hospital	KDIGO	1.0										
Colacchio *et al.* [106]	2023	Italy	R	Surgery (cardiovascular)	RIFLE	2.5	16	69/–/–/31		29				0/0		
Horne *et al.* [107]	2023	UK	P	Hospital	KDIGO	5.0	433	59/24/17/43	1	433	70/70	57/51	70/70	29/29	22/22	
Li *et al.* [108]	2023	China	R	Intrinsic renal disease	KDIGO	4.4	225	39/29/28/61		1 047	28/29	24/15	39/110		0/0	2/2
Nishimoto *et al.* [109]	2023	Japan	R	Surgery (non-cardiovascular)	KDIGO	2.8	69			869	64/63	48/46	89/85	0/0	28/20	35/30
Peerapornratana *et al.* [31]	2023	US	R	Surgery (cardiovascular) & Infection or sepsis	KDIGO	5.0	17 605	20/29/10/40		12 121	63/55	57/58	82/93	0/0	16/9	30/22
Phannajit *et al.* [110]	2023	Thailand	P	Infection or sepsis	KDIGO	4.2	58	22/24/53/78		159						
Xu *et al.* [41]	2023	China	R	Hospital	KDIGO	1.2	11 518	75/16/10/26		54 943	61/59	57/58	96/103	4/4	22/14	40/29
Medunjanin *et al.* [111]	2024	US	R	Hospital	KDIGO	3.0	4 221	69/25/6/31		19 912	75/80	98/98	27/26	100/100		
Zlatanovic *et al.* [112]	2024	Italy	R	Surgery (cardiovascular)	RIFLE	7.6	167	59/25/16/41	7	613	73/69	50/85		41/23	34/18	89/81

BL, baseline; DM, diabetes mellitus; FU, follow-up; HT, hypertension; KRT, kidney replacement therapy; P, prospective; R, retrospective; SCr, serum creatinine.

The ASSESS-AKI studies are from Bhatraju *et al.*, MacLaughlin *et al.*, Ikizler *et al.*, and Hsu *et al.*^40 90 32 91^. Bucaloiu *et al.* included individuals with a recovered kidney function within 90 days. Jones *et al.* included individuals with an AKI duration of <7 days from discharge. Palomba *et al.* included individuals with AKI lasting > 3 days. Yeh *et al.* is categorized as hospital, however, this study focused on community acquired AKI.

a Mean or median is reported, based on what the original study reported.

b Baseline kidney function is reported as eGFR in ml/min/1.73 m^2^ if not stated otherwise. Serum creatinine is expressed in µmol/l.

c 0.3 mg/dl or 50% SCr increase in 4 days;

**Table 2:** Event rates per clinical setting.

						AKI		Non-AKI		
Clinical setting	*N* studies^a^	Mean age (y)	Mean eGFR (ml/min/1.73 m^2^)	Mean percentage males (%)	Mean FU, years [range]^b^	*n* total/*n* outcome	Percentage (%)	*n* total/*n* outcome	Percentage (%)	Relative risk
CKD incidence or CKD progression combined										
Coronary artery disease	1 [35]	72/66	61/79	71/72	8.0	69/48	69.6	911/355	39.0	1.8
Surgery (non-cardiovascular)	2 [64, 82]	67/65	74/75	76/59	1.2 [1–3.2.0]	257/59	23.0	3 220/144	4.5	5.1
Hospital	3 [32, 40, 70, 90, 91, 107]	67/67	68/70	64/56	4.6 [3.0–5.0]	1 340/265	19.8	1 335/86	6.4	3.1
Surgery (cardiovascular)	1 [36]	56/51	86/74 (SCr)	74/59	2.0	1 295/88	6.8	1 950/4	0.2	33.1
CKD incidence only										
Hematologic disease	1 [96]	67/66	84/78	68/54	1.0	324/165	50.9	663/212	32.0	1.6
Surgery (cardiovascular)	4 [34, 37, 38, 39]	60/58	94/97	64/56	2.4 [1.0–3.3]	1 365/363	26.6	2 524/116	4.6	5.8
Infection or sepsis	1 [75]	63/62	85/84	58/56	1.3	358/93	26.0	321/33	10.3	2.5
Hospital	5 [32, 33, 40, 42, 59, 80, 90, 91]	63/51	79/92	72/48	5.4 [1.0–6.0]	37 387/9 209	24.6	1 044 646/84 439	8.1	3.0
CKD progression only										
Hospital	2 [32, 40, 80, 90, 91]	75/65	46/67	49/45	6.0 [4.7–6.0]	5 321/2 134	40.1	118 703/40 934	34.5	1.2
Infection or sepsis	1 [75]	69/70	42/42	52/45	1.3 [1.3–1.3]	297/53	17.8	259/24	9.3	1.9
KF										
Surgery (cardiovascular)	4 [34, 65, 69, 103]	64/62	77/84	76/80	4.4 [3.0–5.2]	1 600/86	5.4	4 198/18	0.4	12.5
Hospital	9 [33, 40, 41, 42, 58, 80, 85, 86, 111] [32, 90, 91]	64/54	74/89	68/49	5.2 [1.0–6.0]	64 336/2 485	3.9	1 244 557/6 529	0.5	7.4
Surgery (non-cardiovascular)	2 [66, 67, 95]	65/65	80/80	59/54	4.1 [3.8–5.0]	14 679/320	2.2	136 353/383	0.3	7.8
Coronary artery disease	3 [35, 61, 87]	70/66	70/75	99/96	1.7 [1.4–8.0]	9 756/22	0.2	20 878/35	0.2	1.3
Infection or sepsis	1 [68]	70/68	74/78	98/97	1.8	13 390/17	0.1	33 067/7	0.0	6.0
Major adverse event										
Coronary artery disease	2 [61, 101]	70/66	70/76	99/98	1.4 [1.0–1.4]	9 674/6 574	68.0	19 282/7 364	38.2	1.8
Infection or sepsis	1 [68]	70/68	74/78	98/97	1.8	13 390/8 262	61.7	33 067/15 825	47.9	1.3
Hospital	2 [32, 40, 42, 90, 91]	62/54	83/91	55/50	2.7 [2.6–4.7]	4 148/1 369	33.0	33 031/5 442	16.5	2.0

Studies are included in this analysis if the number of included participants, event rates, baseline age, percentage of males, and baseline kidney function could be extracted. The mean age, kidney function, percentage of males, and FU duration are weighted based on the study size of included studies.

a Studies of the same cohort are counted as one.

b The weighted mean follow-up, may not be entirely accurate, as studies reported the follow-up duration as a maximum, mean, or median. FU, follow-up; SCr, serum creatinine in µmol/l.

### Kidney failure

**Figure 5:**
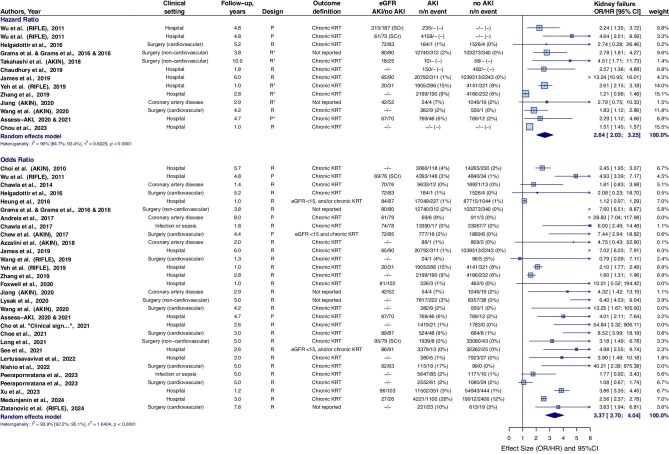
KF in individuals with AKI compared to individuals without AKI. KF occurred in 2.9% (3 666/127 548) of individuals with AKI and in 0.5% (7 560/1 479 866) of individuals without AKI. *Outcome is adjusted for baseline kidney function (eGFR). CI, confidence interval; P, prospective; R, retrospective; SCr, serum creatinine.

KF occurred in 2.9% of individuals with AKI and in 0.5% of individuals without AKI. The pooled HR for KF in individuals with AKI compared to individuals without AKI was 2.64 (95%CI 2.03–3.25), and pooled OR was 3.37 (95%CI 2.70–4.04) (Fig. 5). The higher the AKI stage, the higher the risk for KF. AKI stage 1 showed a pooled HR of 2.55 (95%CI 1.91–3.49) (Fig. S3d). Three studies analyzed the risks of KF in individuals with an AKI duration of <3 days, or 3 days and longer, with pooled OR of 2.59 (95%CI 0.00–2.54) and 3.32 (95%CI 0.00–6.80), respectively [33, 34, 40]. Two studies reported on AKI duration with a cut-off of 7 days, with pooled OR versus individuals without AKI of 2.56 (95%CI 1.00–4.72) for <7 days, and 5.63 (95%CI 4.74–6.52) for AKI of 7 days or longer (mainly weighted by the results of Xu *et al.*) (Fig. S3e) [41, 42]. AKI in the context of cardiovascular surgery showed the highest prevalence of KF (5.4%), and the highest relative risk (12.5) of KF versus individuals without AKI of all included clinical settings (Table 2).

### MAKE

A MAKE occurred in 59.0% of individuals with AKI, and 32.7% of individuals without AKI. Individuals with AKI had a pooled HR of 2.30 (95%CI 2.22–2.39) and pooled OR of 2.77 (95%CI 2.01–3.53) for MAKE, compared to individuals without AKI (Fig. S5). Subanalyses could not be performed due to a lack of available studies.

### Sensitivity analyses

The sensitivity analysis only included studies that reported the outcomes as HRs, studies that defined AKI following the KDIGO criteria, and studies that adjusted the outcomes for baseline kidney function (or studies that had <10% difference between baseline kidney function in individuals without AKI compared to individuals with AKI). The HR in individuals with AKI for CKD incidence was 2.57 (95%CI 2.07–3.07, *I*^2^ = 29.8%), for CKD progression was 2.38 (95%CI 1.48–3.28, *I*^2^ = 0.0%), and for KF was 2.04 (95%CI 1.04–3.02, *I*^2^ = 46.0%), compared to individuals without AKI (Fig. S4).

### Meta-regression

**Figure 6:**
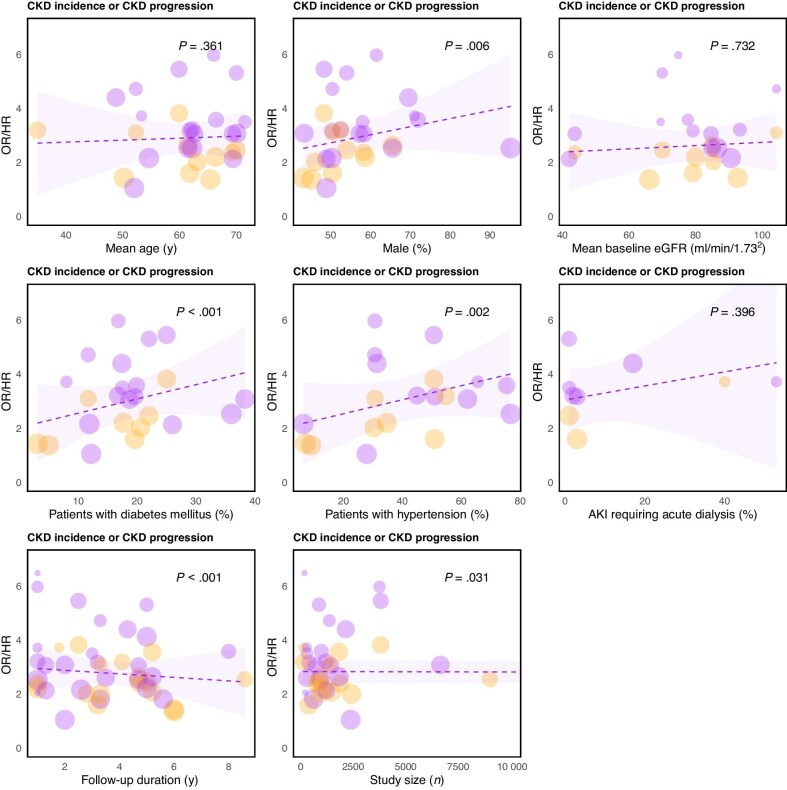
Meta-regression lines to analyze the effect of covariates on the association between AKI and CKD incidence or progression. Every study is represented by a circle. Circle size indicates the study's weight in the random effects model. The line indicates the regression line with 95% confidence intervals. Purple dots represent ORs, orange dots represent HRs. Preferably HRs were included in the meta-regression; however, if HRs were not reported, ORs were used instead. KRT, kidney replacement therapy. *Outcome is adjusted for baseline kidney function (eGFR).

Meta-regression analyses were performed for CKD incidence and progression (and CKD incidence separately) and KF since enough studies could be included for these outcomes. The risk of CKD incidence/progression post-AKI was higher in studies with more men, and when more individuals had diabetes mellitus or hypertension (all *P <* .01). Requiring acute dialysis showed an association with increased CKD incidence/progression risk post-AKI. However, not many studies could be included for this variable, and it showed no significance (*P* = .40) (Fig. 6). The outcomes were comparable in subanalyses for the outcome CKD incidence only (Fig. S6a). KF occurred more in individuals with AKI in studies with longer follow-up (Fig. S6b and c).

## DISCUSSION

This review focused on post-AKI long-term outcomes including CKD incidence, CKD progression, KF, and MAKE, considering the major burden on healthcare posed by these adverse kidney outcomes. Individuals with AKI had higher long-term risks of these outcomes, when compared to individuals without AKI. The risks of long-term kidney dysfunction were higher in individuals with prolonged AKI durations, as well as in specific populations such as individuals with diabetes mellitus, hypertension, individuals requiring acute dialysis for AKI, and individuals undergoing cardiovascular surgery. Importantly, even individuals with the mildest AKI stage, or AKI lasting <3 days, still had a higher risk of kidney dysfunction, compared to individuals without AKI, which may contribute significantly to the global burden of kidney disease, due to high prevalence of mild or short AKI. This review adds to previous systematic reviews on post-AKI adverse kidney events by performing separate meta-analyses for CKD incidence and CKD progression, adding MAKE as outcome of interest, and by performing subgroup analyses on the influence of AKI duration and clinical setting [5, 13, 14, 15].

The importance of AKI duration on mortality is mentioned previously in reviews of Coca *et al.* and Mehta *et al.* [13, 14]. Mehta *et al.* included two studies that studied the risk of CKD incidence, stratified for AKI duration [14]. We were able to expand the insight concerning the association of AKI duration with the outcomes CKD incidence and KF with meta-analyses, as a sufficient number of studies were available for inclusion (eight and five studies, respectively; the four ASSESS-AKI studies counted as one). We found that even AKI lasting <3 days was associated with higher risk of CKD incidence or CKD progression, even in individuals with AKI stage 1. The overall risk for KF was higher in individuals with AKI, nevertheless the subgroup analysis on AKI duration showed that kidney function recovery within 3 days might mitigate this risk to levels similar to those in individuals without AKI. However, the outcome of this meta-analysis might underestimate the risk due to the short follow-up duration (1 year) of the only study that showed no increased risk of KF. Although the pathophysiology is not completely clear, rapid recovery of the kidney function after AKI does not exclude the potential presence of remaining damage [43–45, 46].

Previous research has largely focused on AKI duration and stage as separate factors. However, combining these variables may provide a deeper understanding of their relationship with patient outcomes. In this review, two studies specifically investigated the combined effect of AKI duration and stage on the incidence of CKD [33, 34]. These studies demonstrated that even AKI stage 1 with a duration of 3 days or more was associated with a HR of 2.94 for CKD incidence compared to individuals without AKI. Additionally, Jensen *et al.* analyzed post-AKI outcomes considering both the length of AKI episodes and their severity (without a non-AKI control group). Their results indicate that AKI duration can serve as a valuable complement to AKI stage in predicting prognosis, particularly in cases of mild AKI [47]. Including AKI duration in risk assessments could refine risk stratification and enhance the accuracy of prediction models, leading to better identification of patients at risk for long-term complications.

We found that the prevalence of post-AKI kidney dysfunction varied across different clinical settings, as did the relative risk compared to individuals without AKI. See *et al.* previously showed heterogeneity of pooled results for mortality after AKI between settings [5]. In their analysis, the context of angiography had the largest association with mortality post-AKI. This corresponds with the high prevalence of MAKE (including death) and CKD incidence or progression in the setting of coronary artery disease in our analysis. The highest relative risk for CKD incidence or CKD progression, or KF was in the setting of cardiovascular surgery. AKI in this setting might be caused by several factors such as hemodynamic instability, anesthesia, or postoperative complications such as infections or bleeding [48, 49]. Not all clinical settings were represented in this review. Future research could focus more on the differences in outcomes of AKI based on its causes as we tried by analyzing differences between clinical settings. Diverse causes of AKI may have different consequences for the outcomes.

In clinical practice, substantial underdiagnosis and undertreatment of AKI has been reported [50]. Our review shows the importance of AKI duration on the risk of CKD incidence or progression. This underlines the value of prompt recognition and treatment of AKI. An electronic AKI alert might help recognizing and thus treating AKI in a timely manner [51, 52, 53]. The KDIGO guideline recommends that an individual’s kidney function should be evaluated at 3 months post-AKI to identify the incidence or progression of CKD [21]. However, a recent study showed that only a low percentage of individuals with AKI had their kidney function monitored within 90 days of discharge, and about half of the AKI survivors with CKD were prescribed guideline recommended medications for CKD to prevent cardiorenal consequences [54]. More vigilance regarding kidney function decline post-AKI might be warranted.

This review has its strengths and limitations. A strength of this review is the sensitive search strategy and its ability to consider the heterogeneity of the results by performing subgroup analyses, due to inclusion of a large number of studies. This provides insight into differential risks between individuals with AKI. The inclusion of studies in the meta-analyses was stringent regarding the correct definitions of the outcomes, which enhanced the reliability of the results. A limitation worth noting is that the follow-up duration of the included studies may not always have been sufficiently long to comprehensively assess differences in long-term KF between individuals with and without AKI. This is emphasized by our meta-regression analysis that showed an association between follow-up duration and risk for KF. HRs and ORs were combined in subgroup meta-analyses, although they have slightly distinct risk interpretations. We opted for this approach to use the available data extensively, enhance statistical power, and thereby ensuring a more thorough analysis of the subject. ORs may overestimate risks compared to HRs in studies with a relative short follow-up duration compared to the average time to an event. This discrepancy becomes smaller when the follow-up prolongs. Since we included studies with a minimum mean follow-up duration of one year, we anticipated that the difference would be reduced to a non-clinically significant difference. Further, with regard to the results of Egger's test for funnel plot asymmetry, the results concerning CKD incidence might be influenced by publication bias. The funnel plot asymmetry could also be caused by the heterogeneity of study designs.

## CONCLUSION

AKI was associated with increased risks of kidney dysfunction (CKD incidence, CKD progression, KF, and major adverse kidney outcomes). Risks were heterogeneous between patient subgroups, based on AKI stage, AKI duration, and clinical setting. Yet even the lowest stage or shortest duration of AKI remained at higher risk for CKD incidence or progression. Insight into individual risks for adverse kidney outcomes are necessary to develop tailored follow-up strategies to timely recognize kidney function decline post-AKI and initiate kidney protective measures.

## Supplementary Material

gfaf093_Supplemental_Files

## Data Availability

All data that was used to support the findings of this study were obtained from previously published studies and, therefore, no data are deposited in a public repository. However, the extracted data used for the analyses are available upon request. For inquiries, please contact D.M.J. Veltkamp (d.m.j.veltkamp@umcutrecht.nl).
