# Supplementary material for: Long-term risks of adverse kidney outcomes after acute kidney injury: a systematic review and meta-analysis
Source: Nephrol Dial Transplant. 2025 May 27;40(11):2143–58. doi: 10.1093/ndt/gfaf093 (PMC12559794; doi:10.1093/ndt/gfaf093)
Supplement: gfaf093_Supplemental_Files [file gfaf093_supplemental_files.zip › C.Figures_Supplements_S1_to_S6_20022025.pdf]

Supplementary material including:

- **Figures S1.** Countries of included studies
- **Figure S2.** Funnel plots
- **Figure S3A-E.** Meta-analysis for CKD incidence, CKD progression or Kidney failure in individuals with AKI compared to individuals without AKI, stratified for AKI stage or AKI duration
- **Figure S4.** Meta-analysis for CKD incidence, CKD progression, or Kidney failure in individuals with AKI compared to individuals without AKI; Sensitivity analysis of studies using AKIN and KDIGO AKI criteria, studies with <10% difference in baseline kidney function (or adjusted for baseline kidney function), and studies that reported the outcome as hazard ratio
- **Figure S5.** Major adverse kindey event in individuals with AKI compared to individuals without AKI
- **Figure S6A-C.** Meta-regression analysis to study the effect of covariates on the association between acute kidney injury and CKD incidence (subanalysis) or Kidney Failure

Figure S1. Countries of included studies

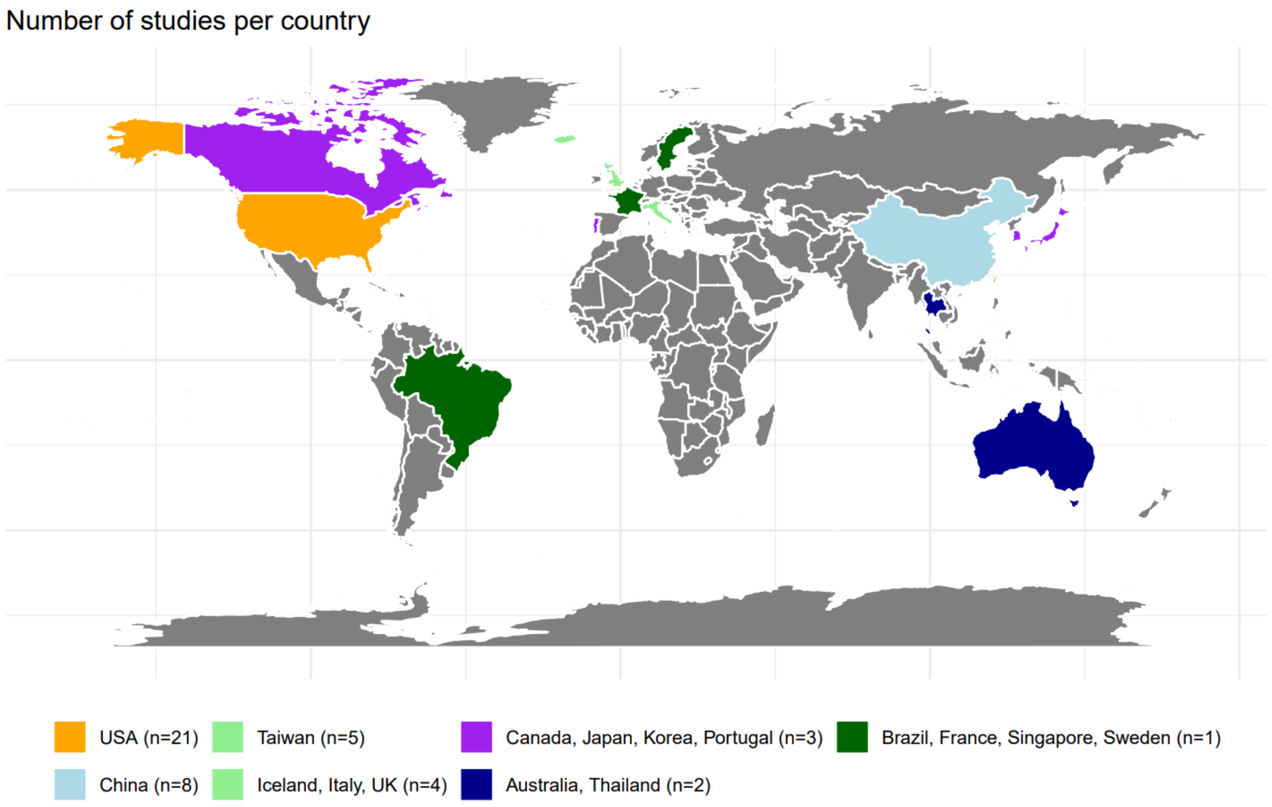

Studies of the same cohort are counted as one

Figure S2. Funnel plots

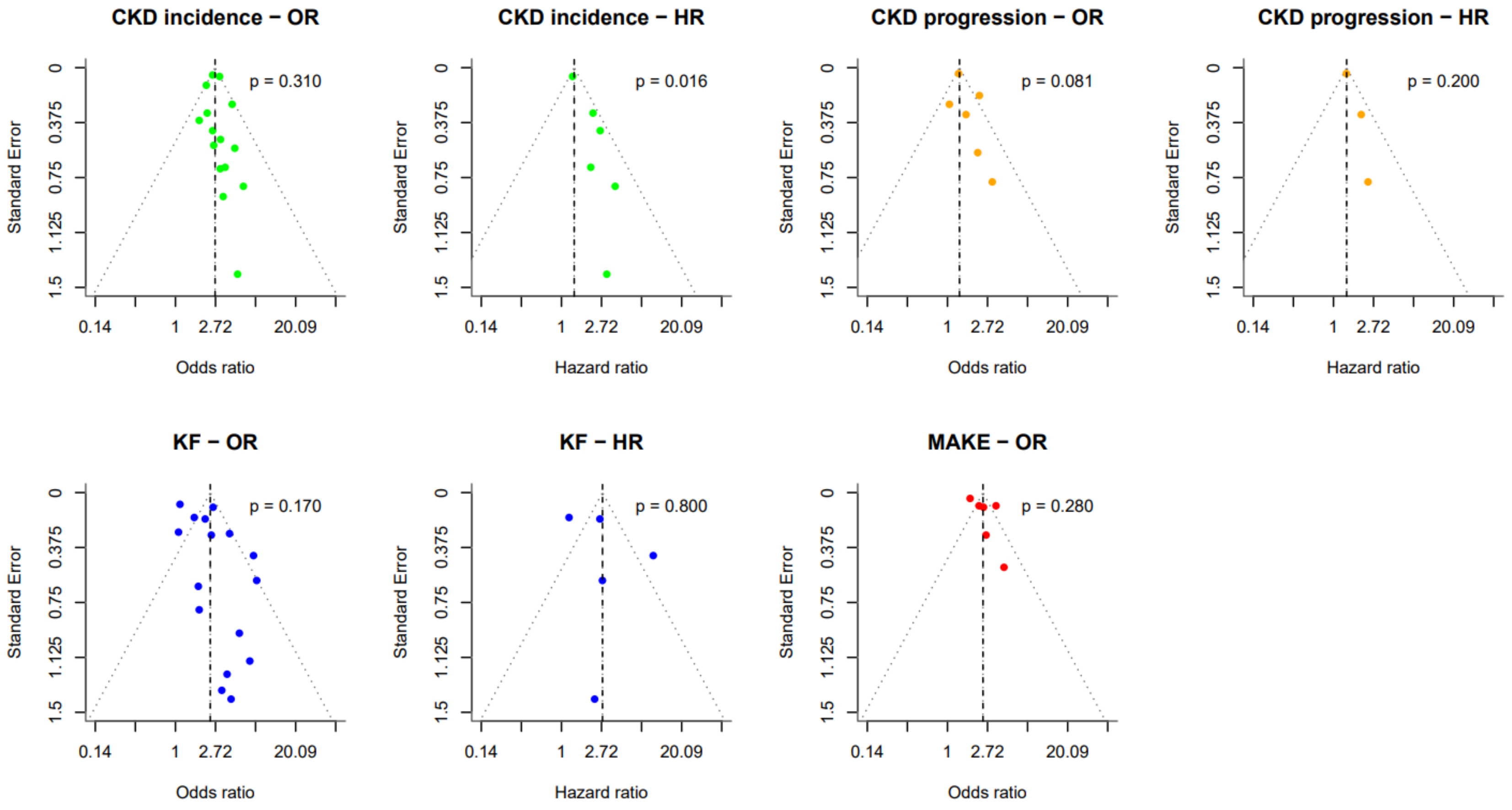

The Egger test for funnel plot asymmetry was used to analyse possible publication bias. The P-values that are shown in the figures derive from the Egger's linear regression test. CKD, chronic kidney disease; HR, Hazard ratio; KF, kidney failure; MAKE, major adverse kidney event; OR, Odds ratio.

Figure S3A. Meta-analysis for CKD incidence or CKD progression in individuals with AKI compared to individuals without AKI, stratified for AKI stage

Authors, Year	Clinical setting	Follow-up, years	Design	Outcome	Outcome definition	eGFR AKI/no AKI	AKI n/n event	no AKI n/n event	Outcome type	CKD incidence or progression OR or HR [95% CI]	weight
**1**											
**Heung et al., 2016**	Hospital	1.0	R	CKD incidence	new eGFR <60	−/87	15566/4774 (31%)	87715/13625 (16%)	OR	2.41 [2.31; 2.50]	10.5%
**Wu et al., 2017**	Surgery (cardiovascular)	3.3	R*	CKD incidence	new eGFR <60	101/105	337/24 (7%)	906/19 (2%)	HR	2.24 [1.21; 4.18]	5.7%
**Neyra et al., 2018**	Infection or sepsis	1.3	R*	CKD incidence	new eGFR <60 + 25% eGFR decrease	81/84	183/42 (23%)	321/33 (10%)	HR	2.13 [1.34; 3.37]	7.5%
**Neyra et al., 2018**	Infection or sepsis	1.3	R	CKD progression	50% eGFR decrease	45/42	181/20 (11%)	259/24 (9%)	OR	1.22 [0.65; 2.28]	8.4%
**Assess−AKI, 2020 & 2021**	Hospital	4.7	P*	CKD incidence and CKD progression	50% eGFR decrease	−/70	566/− (−)	769/− (−)	HR	1.57 [0.91; 2.72]	8.0%
**Cho et al. "Chronic pr..", 2021**	Surgery (cardiovascular)	1.0	R	CKD incidence	new eGFR <60	88/95	−/− (−)	−/− (−)	OR	2.34 [1.31; 4.19]	5.8%
**Cho et al. "Clinical sign...", 2021**	Hospital	2.6	R	CKD incidence	new eGFR <60	−/−	1115/192 (17%)	1651/18 (1%)	OR	18.87 [11.56; 30.80]	0.3%
**Choe et al., 2021**	Surgery (cardiovascular)	3.0	R	CKD incidence	new eGFR <60	89/87	465/216 (46%)	684/56 (8%)	OR	9.73 [7.00; 13.51]	2.0%
**Long et al., 2021**	Surgery (non−cardiovascular)	5.0	R	CKD incidence	new eGFR <60	−/−	347/198 (57%)	26492/6888 (26%)	OR	3.78 [3.05; 4.69]	8.3%
**Long et al., 2021**	Surgery (non−cardiovascular)	5.0	R	CKD progression	1 CKD stage worsening	−/−	351/260 (74%)	6588/3755 (57%)	OR	2.16 [1.69; 2.75]	9.5%
**Menez et al. (AKIN), 2021**	Surgery (cardiovascular)	5.6	R	CKD incidence and CKD progression	50%/25% eGFR decrease + new eGFR <60	−/−	186/64 (34%)	403/96 (24%)	OR	1.68 [1.15; 2.45]	9.0%
**Privratsky et al., 2022**	Surgery (non−cardiovascular)	1.0	R	CKD incidence	new eGFR <60	−/−	135/− (−)	3391/− (−)	HR	5.10 [3.68; 7.07]	5.0%
**Privratsky et al., 2022**	Surgery (non−cardiovascular)	1.0	R	CKD progression	1 CKD stage worsening	−/−	94/− (−)	1134/− (−)	HR	2.83 [1.91; 4.20]	7.0%
**Colacchio et al. (RIFLE), 2023**	Surgery (cardiovascular)	2.5	R	CKD incidence	new eGFR <60	−/−	11/− (−)	29/− (−)	HR	1.36 [0.45; 3.84]	5.0%
**Li et al., 2023**	Intrinsic renal disease	4.4	R*	CKD incidence and CKD progression	50% eGFR decrease	52/110	62/− (−)	727/− (−)	HR	0.68 [0.23; 1.96]	8.1%
**Random effects model**										**2.40 [1.88; 2.92]**	**100.0%**
Heterogeneity: $I^2 = 83.6\%$ [74.3%; 89.5%], $\tau^2 = 0.6664$, $p < 0.0001$											
**2**											
**Heung et al., 2016**	Hospital	1.0	R	CKD incidence	new eGFR <60	−/87	561/206 (37%)	87715/13625 (16%)	OR	3.16 [2.66; 3.75]	28.1%
**Wu et al., 2017**	Surgery (cardiovascular)	3.3	R*	CKD incidence	new eGFR <60	112/105	76/11 (14%)	906/19 (2%)	HR	6.65 [2.93; 15.11]	3.4%
**Assess−AKI, 2020 & 2021**	Hospital	4.7	P*	CKD incidence and CKD progression	50% eGFR decrease	−/70	123/− (−)	769/− (−)	HR	1.67 [0.65; 4.31]	17.6%
**Cho et al. "Chronic pr..", 2021**	Surgery (cardiovascular)	1.0	R	CKD incidence	new eGFR <60	88/95	−/− (−)	−/− (−)	OR	6.11 [2.61; 14.30]	3.7%
**Privratsky et al., 2022**	Surgery (non−cardiovascular)	1.0	R	CKD incidence	new eGFR <60	−/−	27/− (−)	3391/− (−)	HR	3.41 [1.61; 7.22]	11.3%
**Privratsky et al., 2022**	Surgery (non−cardiovascular)	1.0	R	CKD progression	1 CKD stage worsening	−/−	18/− (−)	1134/− (−)	HR	1.91 [0.61; 5.98]	12.0%
**Li et al., 2023**	Intrinsic renal disease	4.4	R*	CKD incidence and CKD progression	50% eGFR decrease	40/110	42/− (−)	727/− (−)	HR	0.96 [0.36; 2.57]	23.8%
**Random effects model**										**2.48 [1.28; 3.68]**	**100.0%**
Heterogeneity: $I^2 = 64.7\%$ [20.4%; 84.3%], $\tau^2 = 1.2525$, $p = 0.0094$											
**3**											
**Heung et al., 2016**	Hospital	1.0	R	CKD incidence	new eGFR <60	−/87	922/439 (48%)	87715/13625 (16%)	OR	4.94 [4.34; 5.63]	72.3%
**Wu et al., 2017**	Surgery (cardiovascular)	3.3	R*	CKD incidence	new eGFR <60	100/105	44/7 (16%)	906/19 (2%)	HR	24.19 [8.65; 67.66]	0.1%
**Assess−AKI, 2020 & 2021**	Hospital	4.7	P*	CKD incidence and CKD progression	50% eGFR decrease	−/70	80/− (−)	769/− (−)	HR	3.42 [1.28; 9.12]	5.9%
**Cho et al. "Chronic pr..", 2021**	Surgery (cardiovascular)	1.0	R	CKD incidence	new eGFR <60	88/95	−/− (−)	−/− (−)	OR	10.19 [2.94; 35.34]	0.4%
**Privratsky et al., 2022**	Surgery (non−cardiovascular)	1.0	R	CKD incidence	new eGFR <60	−/−	23/− (−)	3391/− (−)	HR	3.32 [1.37; 8.05]	8.0%
**Privratsky et al., 2022**	Surgery (non−cardiovascular)	1.0	R	CKD progression	1 CKD stage worsening	−/−	7/− (−)	1134/− (−)	HR	7.02 [2.23; 22.04]	1.0%
**Li et al., 2023**	Intrinsic renal disease	4.4	R*	CKD incidence and CKD progression	50% eGFR decrease	24/110	51/− (−)	727/− (−)	HR	2.52 [1.01; 6.28]	12.3%
**Random effects model**										**4.48 [3.50; 5.47]**	**100.0%**
Heterogeneity: $I^2 = 8.2\%$ [0.0%; 73.2%], $\tau^2 = 0.2397$, $p = 0.3662$											
**2 or 3**											
**Neyra et al., 2018**	Infection or sepsis	1.3	R*	CKD incidence	new eGFR <60 + 25% eGFR decrease	88/84	175/51 (29%)	321/33 (10%)	HR	2.79 [1.79; 4.34]	25.3%
**Cho et al. "Clinical sign...", 2021**	Hospital	2.6	R	CKD incidence	new eGFR <60	−/−	57/16 (28%)	1651/18 (1%)	OR	35.40 [16.87; 74.30]	0.3%
**Choe et al., 2021**	Surgery (cardiovascular)	3.0	R	CKD incidence	new eGFR <60	90/87	59/43 (73%)	684/56 (8%)	OR	30.14 [15.96; 56.91]	0.5%
**Long et al., 2021**	Surgery (non−cardiovascular)	5.0	R	CKD incidence	new eGFR <60	−/−	828/497 (60%)	26492/6888 (26%)	OR	4.27 [3.71; 4.92]	29.5%
**Long et al., 2021**	Surgery (non−cardiovascular)	5.0	R	CKD progression	1 CKD stage worsening	−/−	413/310 (75%)	6588/3755 (57%)	OR	2.27 [1.81; 2.85]	29.9%
**Menez et al. (AKIN), 2021**	Surgery (cardiovascular)	5.6	R	CKD incidence and CKD progression	50%/25% eGFR decrease + new eGFR <60	−/−	24/12 (50%)	403/96 (24%)	OR	3.20 [1.39; 7.35]	14.0%
**Colacchio et al. (RIFLE), 2023**	Surgery (cardiovascular)	2.5	R	CKD incidence	new eGFR <60	−/−	5/− (−)	29/− (−)	HR	10.25 [2.21; 43.73]	0.5%
**Random effects model**										**3.40 [1.90; 4.91]**	**100.0%**
Heterogeneity: $I^2 = 83.5\%$ [67.5%; 91.6%], $\tau^2 = 1.9030$, $p < 0.0001$											

Heterogeneity: $I^2 = 83.8\%$ [78.4%; 87.8%], $\tau^2 = 0.8964$, $p < 0.0001$
Test for subgroup differences: $\chi^2_3 = 14.38$, df = 3 ($p = 0.0024$)

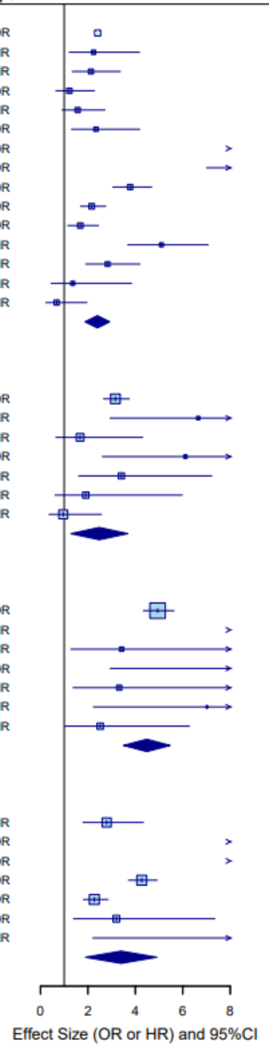

AKI, acute kidney injury; CI, confidence interval; CKD, chronic kidney disease; HR, hazard ratio; OR, odds ratio. If a HR was reported, then the HR is included. Otherwise the outcome is reported as OR. *Outcome is adjusted for baseline kidney function

Figure S3B. Meta-analysis for CKD incidence (subanalysis) in individuals with AKI compared to individuals without AKI, stratified for AKI stage

Authors, Year	Clinical setting	Follow-up, years	Design	Outcome definition	eGFR AKI/no AKI	AKI n/n event	no AKI n/n event	Outcome type	CKD incidence OR or HR [95% CI]	weight
**1**										
**Heung et al., 2016**	Hospital	1.0	R	new eGFR <60	−/87	15566/4774 (31%)	87715/13625 (16%)	OR	2.41 [2.31; 2.50]	17.0%
**Wu et al., 2017**	Surgery (cardiovascular)	3.3	R*	new eGFR <60	101/105	337/24 (7%)	906/19 (2%)	HR	2.24 [1.21; 4.18]	12.1%
**Neyra et al., 2018**	Infection or sepsis	1.3	R*	new eGFR <60 + 25% eGFR decrease	81/84	183/42 (23%)	321/33 (10%)	HR	2.13 [1.34; 3.37]	14.3%
**Cho et al. "Chronic pr..", 2021**	Surgery (cardiovascular)	1.0	R	new eGFR <60	88/95	−/− (−)	−/− (−)	OR	2.34 [1.31; 4.19]	12.3%
**Cho et al. "Clinical sign…", 2021**	Hospital	2.6	R	new eGFR <60	−/−	1115/192 (17%)	1651/18 (1%)	OR	18.87 [11.56; 30.80]	0.9%
**Choe et al., 2021**	Surgery (cardiovascular)	3.0	R	new eGFR <60	89/87	465/216 (46%)	684/56 (8%)	OR	9.73 [7.00; 13.51]	5.8%
**Long et al., 2021**	Surgery (non−cardiovascular)	5.0	R	new eGFR <60	−/−	347/198 (57%)	26492/6888 (26%)	OR	3.78 [3.05; 4.69]	15.2%
**Privratsky et al., 2022**	Surgery (non−cardiovascular)	1.0	R	new eGFR <60	−/−	135/− (−)	3391/− (−)	HR	5.10 [3.68; 7.07]	11.2%
**Colacchio et al. (RIFLE), 2023**	Surgery (cardiovascular)	2.5	R	new eGFR <60	−/−	11/− (−)	29/− (−)	HR	1.36 [0.45; 3.84]	11.2%
**Random effects model**									**3.31 [2.35; 4.27]**	**100.0%**
Heterogeneity: I^2 = 84.8% [72.9%; 91.5%], τ^2 = 1.4146, $p < 0.0001$										
**2**										
**Heung et al., 2016**	Hospital	1.0	R	new eGFR <60	−/87	561/206 (37%)	87715/13625 (16%)	OR	3.16 [2.66; 3.75]	94.8%
**Wu et al., 2017**	Surgery (cardiovascular)	3.3	R*	new eGFR <60	112/105	76/11 (14%)	906/19 (2%)	HR	6.65 [2.93; 15.11]	0.8%
**Cho et al. "Chronic pr..", 2021**	Surgery (cardiovascular)	1.0	R	new eGFR <60	88/95	−/− (−)	−/− (−)	OR	6.11 [2.61; 14.30]	0.8%
**Privratsky et al., 2022**	Surgery (non−cardiovascular)	1.0	R	new eGFR <60	−/−	27/− (−)	3391/− (−)	HR	3.41 [1.61; 7.22]	3.6%
**Random effects model**									**3.22 [2.69; 3.75]**	**100.0%**
Heterogeneity: I^2 = 0% [0.0%; 84.7%], τ^2 = 0, p = 0.5277										
**3**										
**Heung et al., 2016**	Hospital	1.0	R	new eGFR <60	−/87	922/439 (48%)	87715/13625 (16%)	OR	4.94 [4.34; 5.63]	96.2%
**Wu et al., 2017**	Surgery (cardiovascular)	3.3	R*	new eGFR <60	100/105	44/7 (16%)	906/19 (2%)	HR	24.19 [8.65; 67.66]	0.0%
**Cho et al. "Chronic pr..", 2021**	Surgery (cardiovascular)	1.0	R	new eGFR <60	88/95	−/− (−)	−/− (−)	OR	10.19 [2.94; 35.34]	0.2%
**Privratsky et al., 2022**	Surgery (non−cardiovascular)	1.0	R	new eGFR <60	−/−	23/− (−)	3391/− (−)	HR	3.32 [1.37; 8.05]	3.6%
**Random effects model**									**4.90 [4.27; 5.53]**	**100.0%**
Heterogeneity: I^2 = 0% [0.0%; 84.7%], τ^2 = 0, p = 0.4032										
**2 or 3**										
**Neyra et al., 2018**	Infection or sepsis	1.3	R*	new eGFR <60 + 25% eGFR decrease	88/84	175/51 (29%)	321/33 (10%)	HR	2.79 [1.79; 4.34]	45.8%
**Cho et al. "Clinical sign…", 2021**	Hospital	2.6	R	new eGFR <60	−/−	57/16 (28%)	1651/18 (1%)	OR	35.40 [16.87; 74.30]	0.7%
**Choe et al., 2021**	Surgery (cardiovascular)	3.0	R	new eGFR <60	90/87	59/43 (73%)	684/56 (8%)	OR	30.14 [15.96; 56.91]	1.4%
**Long et al., 2021**	Surgery (non−cardiovascular)	5.0	R	new eGFR <60	−/−	828/497 (60%)	26492/6888 (26%)	OR	4.27 [3.71; 4.92]	50.8%
**Colacchio et al. (RIFLE), 2023**	Surgery (cardiovascular)	2.5	R	new eGFR <60	−/−	5/− (−)	29/− (−)	HR	10.25 [2.21; 43.73]	1.3%
**Random effects model**									**4.25 [1.82; 6.68]**	**100.0%**
Heterogeneity: I^2 = 74.1% [35.6%; 89.6%], τ^2 = 2.9296, p = 0.0039										

Heterogeneity: I^2 = 87.4% [82.2%; 91.0%], τ^2 = 1.4832, $p < 0.0001$

Test for subgroup differences: χ^2_3 = 17.27, df = 3 (p = 0.0006)

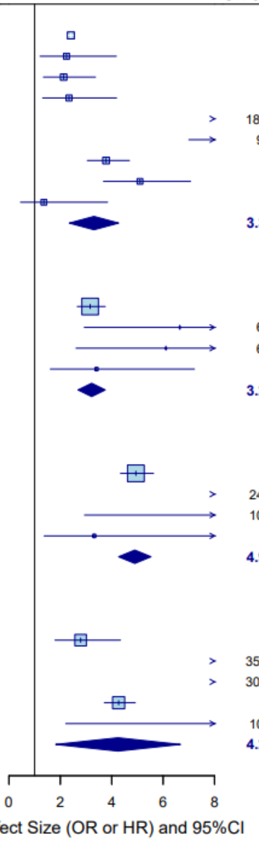

AKI, acute kidney injury; CI, confidence interval; CKD, chronic kidney disease; HR, hazard ratio; OR, odds ratio. If a HR was reported, then the HR is included. Otherwise the outcome is reported as OR. *Outcome is adjusted for baseline kidney function

Figure S3C. Meta-analysis for CKD progression (subanalysis) in individuals with AKI compared to individuals without AKI, stratified for AKI stage

Authors, Year	Clinical setting	Follow−up, years	Design	Outcome definition	eGFR AKI/no AKI	AKI n/n event	no AKI n/n event	Outcome type	CKD progression OR or HR [95% CI]	weight
**1**										
**Neyra et al., 2018**	Infection or sepsis	1.3	R	50% eGFR decrease	45/42	181/20 (11%)	259/24 (9%)	OR	1.22 [0.65; 2.28]	33.3%
**Long et al., 2021**	Surgery (non−cardiovascular)	5.0	R	1 CKD stage worsening	−/−	351/260 (74%)	6588/3755 (57%)	OR	2.16 [1.69; 2.75]	41.8%
**Privratsky et al., 2022**	Surgery (non−cardiovascular)	1.0	R	1 CKD stage worsening	−/−	94/− (−)	1134/− (−)	HR	2.83 [1.91; 4.20]	24.8%
**Random effects model**									**2.01 [1.22; 2.81]**	**100.0%**
Heterogeneity: $I^2 = 66\%$ [0.0%; 90.2%], $\tau^2 = 0.3197$, $p = 0.0527$										
**2 or 3**										
**Neyra et al., 2018**	Infection or sepsis	1.3	R	50% eGFR decrease	40/42	116/33 (28%)	259/24 (9%)	OR	3.89 [2.17; 6.97]	4.3%
**Long et al., 2021**	Surgery (non−cardiovascular)	5.0	R	1 CKD stage worsening	−/−	413/310 (75%)	6588/3755 (57%)	OR	2.27 [1.81; 2.85]	92.0%
**Privratsky et al., 2022**	Surgery (non−cardiovascular)	1.0	R	1 CKD stage worsening	−/−	18/− (−)	1134/− (−)	HR	1.91 [0.61; 5.98]	3.4%
**Privratsky et al., 2022**	Surgery (non−cardiovascular)	1.0	R	1 CKD stage worsening	−/−	7/− (−)	1134/− (−)	HR	7.02 [2.23; 22.04]	0.3%
**Random effects model**									**2.34 [1.84; 2.84]**	**100.0%**
Heterogeneity: $I^2 = 0\%$ [0.0%; 84.7%], $\tau^2 = 0$, $p = 0.4526$										

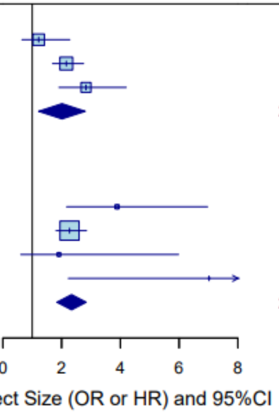

Heterogeneity: $I^2 = 37.1\%$ [0.0%; 73.5%], $\tau^2 = 0.1339$, $p = 0.1455$

Test for subgroup differences: $\chi^2_1 = 0.47$, df = 1 ($p = 0.4950$)

AKI, acute kidney injury; CI, confidence interval; CKD, chronic kidney disease; HR, hazard ratio; OR, odds ratio. If a HR was reported, then the HR is included. Otherwise the outcome is reported as OR.

Figure S3D. Meta-analysis for Kidney failure in individuals with AKI compared to individuals without AKI, stratified for AKI stage

Authors, Year	Clinical setting	Follow−up, years	Design	Outcome	Outcome definition	eGFR AKI/no AKI	AKI n/n event	no AKI n/n event	Outcome type	Kidney failure HR [95% CI]	weight
**1**											
**Choi et al. (AKIN), 2010**	Hospital	5.7	R*	Kidney failure	Chronic KRT	−/−	2453/63 (3%)	14265/230 (2%)	HR	1.37 [1.02; 1.84]	28.3%
**James et al. (AKIN), 2011**	Coronary artery disease	1.6	R*	Kidney failure	Chronic KRT	67/75	−/− (−)	−/− (−)	HR	4.15 [2.32; 7.42]	9.4%
**Wu et al. (RIFLE), 2011**	Hospital	4.8	P	Kidney failure	Chronic KRT	−/−	2434/14 (1%)	4724/13 (0%)	HR	2.09 [0.97; 4.52]	14.5%
**Rydén et al. (AKIN), 2014**	Surgery (cardiovascular)	4.3	R*	Kidney failure	Chronic KRT	69/78	3159/50 (2%)	25609/44 (0%)	HR	2.92 [1.87; 4.55]	18.6%
**Grams et al. & Grams et al., 2016 & 2016**	Surgery (non−cardiovascular)	3.8	R*	Kidney failure	Not reported	−/−	10238/211 (2%)	103273/340 (0%)	HR	2.53 [1.57; 4.09]	19.5%
**Chew et al. (AKIN), 2017**	Surgery (cardiovascular)	4.4	P	Kidney failure	eGFR <15 and chronic KRT	72/85	608/12 (2%)	1889/6 (0%)	HR	4.70 [1.74; 12.60]	2.7%
**Assess−AKI, 2020 & 2021**	Hospital	4.7	P	Kidney failure	Chronic KRT	−/70	566/− (−)	769/− (−)	HR	4.39 [2.27; 8.51]	7.0%
**Random effects model**										**2.55 [1.61; 3.49]**	**100.0%**
Heterogeneity: I^2 = 60.2% [8.8%; 82.7%], τ^2 = 0.7724, p = 0.0195											
**2**											
**Wu et al. (RIFLE), 2011**	Hospital	4.8	P	Kidney failure	Chronic KRT	−/−	979/7 (1%)	4724/13 (0%)	HR	3.19 [1.27; 8.03]	44.6%
**Grams et al. & Grams et al., 2016 & 2016**	Surgery (non−cardiovascular)	3.8	R*	Kidney failure	Not reported	−/−	1687/42 (2%)	103273/340 (0%)	HR	3.63 [1.31; 10.00]	27.0%
**Assess−AKI, 2020 & 2021**	Hospital	4.7	P	Kidney failure	Chronic KRT	−/70	123/− (−)	769/− (−)	HR	3.31 [1.14; 9.60]	28.5%
**Random effects model**										**3.34 [1.09; 5.60]**	**100.0%**
Heterogeneity: I^2 = 0% [0.0%; 89.6%], τ^2 = 0, p = 0.9876											
**3**											
**Wu et al. (RIFLE), 2011**	Hospital	4.8	P	Kidney failure	Chronic KRT	−/−	745/58 (8%)	4724/13 (0%)	HR	22.35 [11.90; 42.10]	19.1%
**Grams et al. & Grams et al., 2016 & 2016**	Surgery (non−cardiovascular)	3.8	R*	Kidney failure	Not reported	−/−	815/59 (7%)	103273/340 (0%)	HR	2.62 [0.48; 14.30]	39.6%
**Assess−AKI, 2020 & 2021**	Hospital	4.7	P	Kidney failure	Chronic KRT	−/70	80/− (−)	769/− (−)	HR	5.76 [2.21; 14.99]	41.3%
**Random effects model**										**7.68 [−0.47; 15.83]**	**100.0%**
Heterogeneity: I^2 = 63.1% [0.0%; 89.5%], τ^2 = 31.1982, p = 0.0663											
**2 or 3**											
**Choi et al. (AKIN), 2010**	Hospital	5.7	R*	Kidney failure	Chronic KRT	−/−	273/23 (8%)	14265/230 (2%)	HR	3.80 [2.32; 6.23]	32.8%
**Choi et al. (AKIN), 2010**	Hospital	5.7	R*	Kidney failure	Chronic KRT	−/−	334/32 (10%)	14265/230 (2%)	HR	20.36 [12.65; 32.78]	9.5%
**James et al. (AKIN), 2011**	Coronary artery disease	1.6	R*	Kidney failure	Chronic KRT	59/75	−/− (−)	−/− (−)	HR	11.74 [6.38; 21.59]	13.9%
**Rydén et al. (AKIN), 2014**	Surgery (cardiovascular)	4.3	R*	Kidney failure	Chronic KRT	71/78	562/29 (5%)	25609/44 (0%)	HR	3.81 [2.14; 6.79]	31.6%
**Chew et al. (AKIN), 2017**	Surgery (cardiovascular)	4.4	P	Kidney failure	eGFR <15 and chronic KRT	72/85	169/6 (4%)	1889/6 (0%)	HR	5.80 [1.77; 18.73]	12.1%
**Random effects model**										**6.73 [3.11; 10.35]**	**100.0%**
Heterogeneity: I^2 = 71.3% [27.4%; 88.7%], τ^2 = 9.3768, p = 0.0075											

Heterogeneity: I^2 = 67.6% [47.1%; 80.2%], τ^2 = 2.2081, p < 0.0001
Test for subgroup differences: χ^2_3 = 6.29, df = 3 (p = 0.0981)

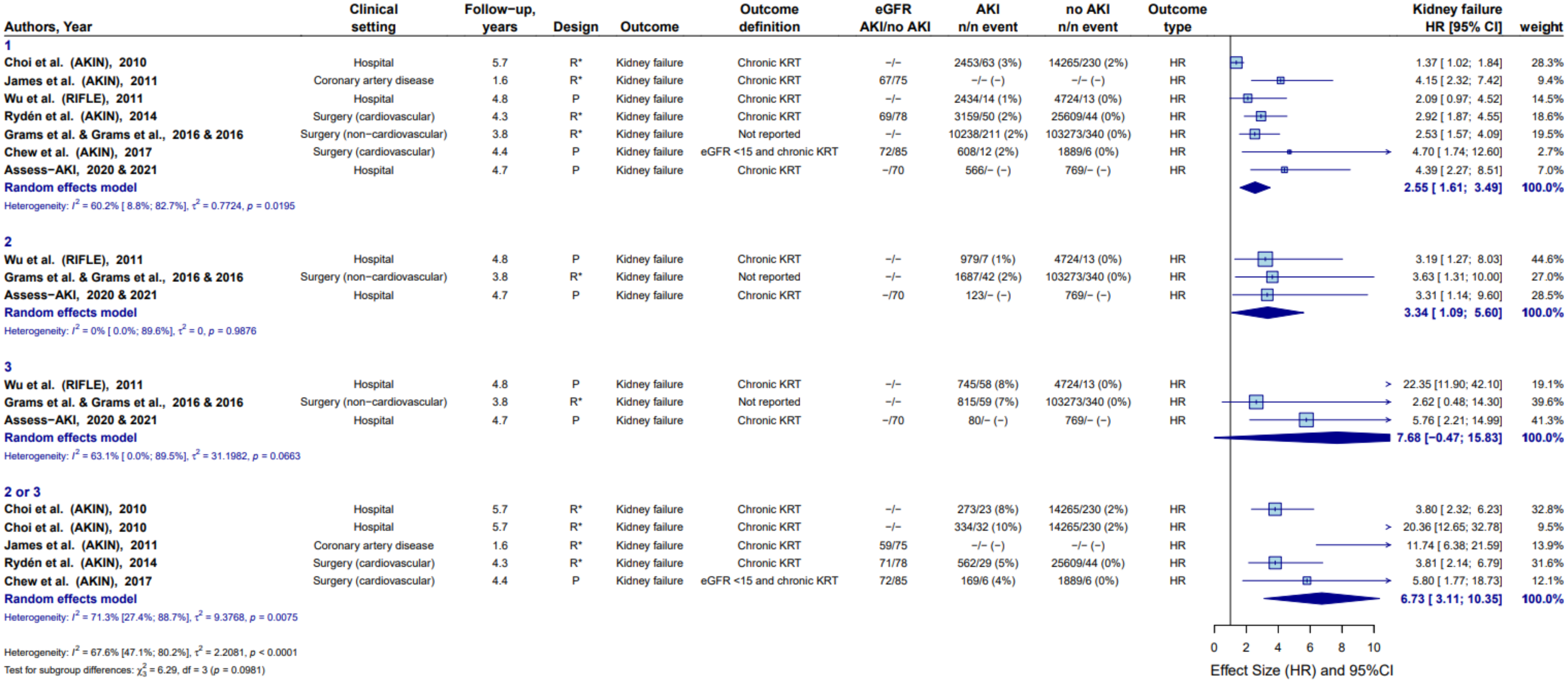

HRs shown only. AKI, acute kidney injury; CI, confidence interval; HR, hazard ratio; KF, kidney failure. *Outcome is adjusted for baseline kidney function. Choi et al. with HR 20.36 included individuals with AKI requiring acute kidney replacement therapy only.

Figure S3E. Meta-analysis for Kidney failure in individuals with AKI compared to individuals without AKI, stratified for AKI duration

Authors, Year	Clinical setting	Follow-up, years	Design	Outcome definition	AKI n/n event	no AKI n/n event	CKD incidence OR [95% CI]	weight
**<3 days**								
Heung et al., 2016	Hospital	1.0	R	eGFR <15, and/or chronic KRT	12072/127 (1%)	87715/1044 (1%)	0.88 [0.73; 1.06]	48.0%
Assess−AKI, 2020 & 2021	Hospital	4.7	P	Chronic KRT	475/28 (6%)	769/12 (2%)	3.95 [1.99; 7.85]	30.3%
Choe et al., 2021	Surgery (cardiovascular)	3.0	R	Chronic KRT	376/19 (5%)	684/8 (1%)	4.50 [1.95; 10.38]	21.7%
**Random effects model**							**2.59 [−0.05; 5.24]**	**100.0%**
Heterogeneity: $I^2 = 71.5\%$ [3.3%; 91.6%], $\tau^2 = 3.7942$, $p = 0.0299$								
**3 or more days**								
Heung et al., 2016	Hospital	1.0	R	eGFR <15, and/or chronic KRT	3946/84 (2%)	87715/1044 (1%)	1.81 [1.44; 2.26]	58.5%
Assess−AKI, 2020 & 2021	Hospital	4.7	P	Chronic KRT	294/18 (6%)	769/12 (2%)	4.11 [1.96; 8.65]	38.2%
Choe et al., 2021	Surgery (cardiovascular)	3.0	R	Chronic KRT	148/29 (20%)	684/8 (1%)	20.59 [9.19; 46.13]	3.4%
**Random effects model**							**3.32 [−0.17; 6.80]**	**100.0%**
Heterogeneity: $I^2 = 65.2\%$ [0.0%; 90.0%], $\tau^2 = 5.3595$, $p = 0.0566$								
**<7 days**								
See et al., 2021	Hospital	2.6	R	eGFR <15, and/or chronic KRT	2596/9 (0%)	32262/25 (0%)	4.49 [2.09; 9.62]	14.5%
Xu et al., 2023	Hospital	1.2	R	Chronic KRT	5879/105 (2%)	54943/444 (1%)	2.23 [1.80; 2.77]	85.5%
**Random effects model**							**2.56 [1.00; 4.12]**	**100.0%**
Heterogeneity: $I^2 = 26.6\%$, $\tau^2 = 0.6782$, $p = 0.2433$								
**7 or more days**								
See et al., 2021	Hospital	2.6	R	eGFR <15, and/or chronic KRT	783/4 (1%)	32262/25 (0%)	6.62 [2.30; 19.07]	1.1%
Xu et al., 2023	Hospital	1.2	R	Chronic KRT	5623/246 (4%)	54943/444 (1%)	5.62 [4.79; 6.58]	98.9%
**Random effects model**							**5.63 [4.74; 6.52]**	**100.0%**
Heterogeneity: $I^2 = 0\%$, $\tau^2 = 0$, $p = 0.8162$								

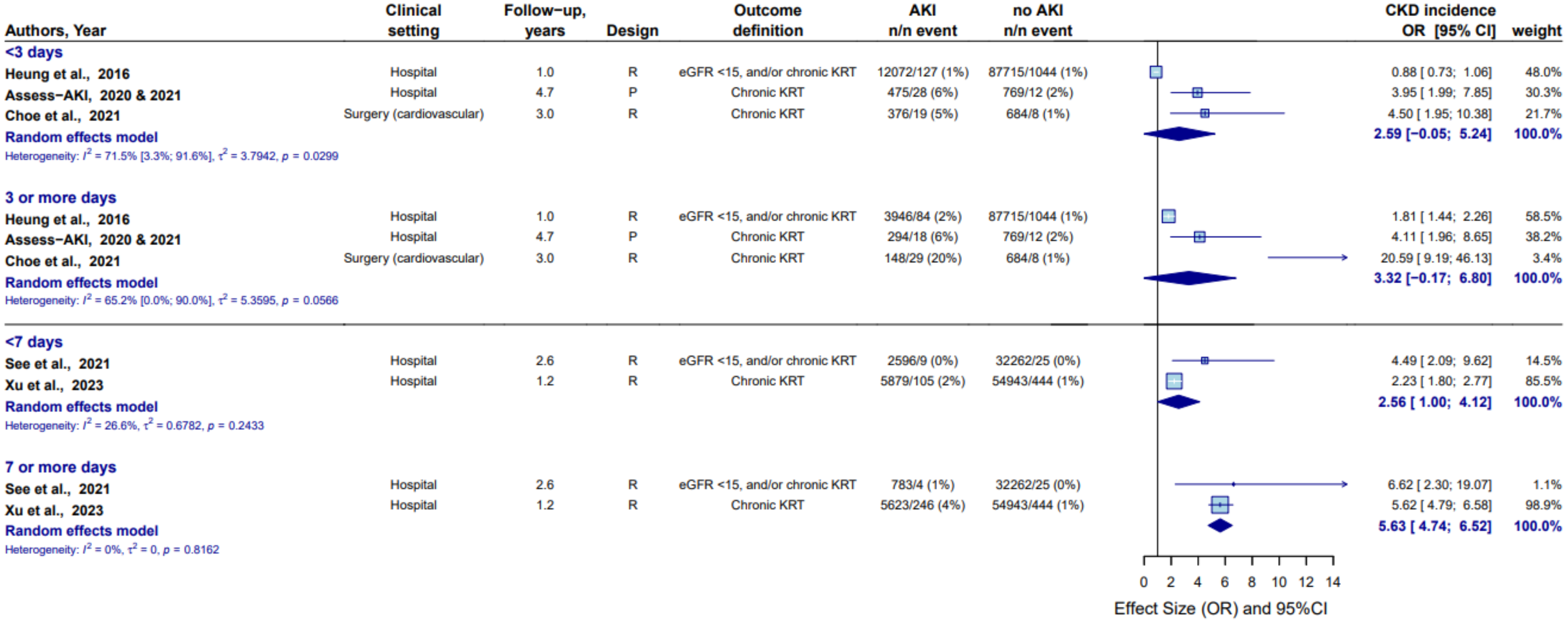

ORs shown only (only See et al. and the Assess-AKI studies reported hazard ratios). AKI, acute kidney injury; CI, confidence interval; OR, odds ratio.

Figure S4. Meta-analysis for CKD incidence, CKD progression, or Kidney failure in individuals with AKI compared to individuals without AKI; Sensitivity analysis of studies using AKIN and KDIGO AKI criteria, studies with <10% difference in baseline kidney function (or adjusted for baseline kidney function), and studies that reported the outcome as hazard ratio

Authors, Year	Clinical setting	Follow-up, years	Design	Outcome definition	eGFR AKI/no AKI	AKI n/n event	no AKI n/n event	HR [95% CI]	weight
**CKD incidence and CKD progression**									
Thakar et al., 2011	Hospital	5.2	R*	new eGFR <30	94/99 (SCr)	530/124 (23%)	1292/134 (10%)	3.56 [2.76; 4.61]	23.5%
Gameiro et al., 2016	Surgery (non−cardiovascular)	3.2	R*	25% eGFR decrease	80/79	72/34 (47%)	318/70 (22%)	1.60 [1.01; 2.50]	26.1%
Zhang et al., 2018	Intrinsic renal disease	4.1	R*	50% eGFR decrease	116/76 (SCr)	−/− (−)	−/− (−)	3.19 [2.05; 4.96]	16.6%
Assess−AKI, 2020 & 2021	Hospital	4.7	P*	50% eGFR decrease	67/70	769/97 (13%)	769/41 (5%)	2.53 [1.21; 5.25]	11.4%
Horne et al., 2023	Hospital	5.0	P*	50% eGFR decrease	70/70	421/138 (33%)	416/35 (8%)	2.46 [1.66; 3.65]	22.5%
**Random effects model**								**2.62 [1.78; 3.47]**	**100.0%**
Heterogeneity: I^2 = 65.3% [9.1%; 86.7%], τ^2 = 0.5689, p = 0.0213									
**CKD incidence**									
Jones et al., 2012	Hospital	2.5	R*	new eGFR <60	80/80 (SCr)	719/108 (15%)	3090/97 (3%)	3.82 [2.81; 5.19]	13.6%
Sawhney et al., 2017	Hospital	8.6	R*	new eGFR <30	−/−	955/− (−)	8049/− (−)	2.55 [1.41; 4.64]	8.3%
Wu et al., 2017	Surgery (cardiovascular)	3.3	R*	new eGFR <60	103/105	457/42 (9%)	906/19 (2%)	3.10 [1.75; 5.48]	6.4%
Assess−AKI, 2020 & 2021	Hospital	4.7	P*	new eGFR <60 + 25% eGFR decrease	84/86	463/137 (30%)	463/66 (14%)	2.63 [1.91; 3.61]	21.7%
May et al., 2021	Hematologic disease	1.0	R	new eGFR <60	84/78	324/165 (51%)	663/212 (32%)	2.20 [1.70; 2.80]	34.1%
Nishimoto et al., 2023	Surgery (non−cardiovascular)	2.8	R*	new eGFR <60	89/85	−/− (−)	−/− (−)	2.02 [1.21; 3.35]	15.9%
**Random effects model**								**2.57 [2.07; 3.07]**	**100.0%**
Heterogeneity: I^2 = 29.8% [0.0%; 71.4%], τ^2 = 0.1136, p = 0.2113									
**CKD progression**									
Assess−AKI, 2020 & 2021	Hospital	4.7	P*	50% eGFR decrease	42/46	306/70 (23%)	306/27 (9%)	2.37 [1.28; 4.39]	33.6%
Go et al., 2021	Coronary artery disease	1.0	R*	50% eGFR decrease	−/−	188/− (−)	1690/− (−)	2.39 [1.53; 3.74]	66.4%
**Random effects model**								**2.38 [1.48; 3.28]**	**100.0%**
Heterogeneity: I^2 = 0%, τ^2 = 0, p = 0.9836									
**Kidney failure**									
Helgadottir et al., 2016	Surgery (cardiovascular)	5.2	R	Chronic KRT	72/83	184/1 (1%)	1526/4 (0%)	2.74 [0.28; 26.46]	0.6%
Grams et al. & Grams et al., 2016 & 2016	Surgery (non−cardiovascular)	3.8	R*	Not reported	80/80	12740/312 (2%)	103273/340 (0%)	2.78 [1.81; 4.27]	27.3%
Takahashi et al. (AKIN), 2018	Surgery (non−cardiovascular)	10.0	R*	Chronic KRT	18/25	10/− (−)	58/− (−)	4.51 [1.71; 11.73]	3.6%
Zhang et al., 2019	Hospital	2.8	R*	Chronic KRT	−/−	2199/190 (9%)	4166/232 (6%)	1.21 [0.98; 1.48]	45.7%
Jiang (AKIN), 2020	Coronary artery disease	2.9	R*	Not reported	42/52	54/4 (7%)	1046/19 (2%)	2.79 [0.75; 10.33]	3.9%
Assess−AKI, 2020 & 2021	Hospital	4.7	P*	Chronic KRT	67/70	769/46 (6%)	769/12 (2%)	2.29 [1.12; 4.66]	18.8%
**Random effects model**								**2.03 [1.04; 3.02]**	**100.0%**
Heterogeneity: I^2 = 46% [0.0%; 78.6%], τ^2 = 0.5430, p = 0.099									

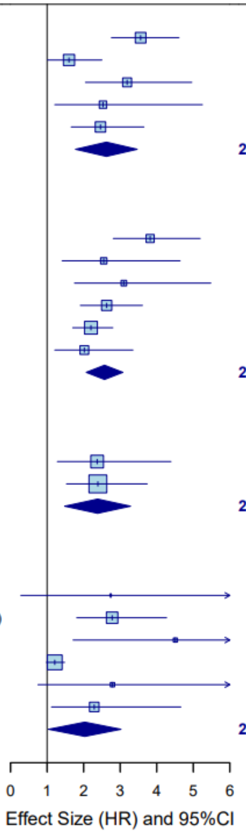

AKI, acute kidney injury; AKIN, Acute kidney injury network ;CI, confidence interval; CKD, chronic kidney disease, HR, hazard ratio

Figure S5. Major adverse kindey event in individuals with AKI compared to individuals without AKI

Authors, Year	Clinical setting	Follow−up, years	Design	MAKE definition	eGFR AKI/no AKI	AKI n/n event	no AKI n/n event	MAKE OR or HR [95% CI]	weight
**Hazard Ratio**									
**Chawla et al., 2014**	Coronary artery disease	1.4	R*	Death, chronic KRT, 25% eGFR decrease	70/76	9633/6558 (68%)	18921/7351 (39%)	2.30 [2.21; 2.38]	99.6%
**Chen, L. et al. (AKIN), 2022**	Coronary artery disease	1.0	P	Death, chronic KRT, 50% SCr increase	80/91	41/16 (39%)	361/13 (4%)	11.58 [4.29; 31.24]	0.0%
**Phannajit et al., 2023**	Infection or sepsis	4.2	P	Death, chronic KRT, new eGFR <60, tubulopathy	−/−	58/− (−)	159/− (−)	2.26 [1.28; 4.00]	0.4%
**Random effects model**								**2.30 [2.22; 2.39]**	**100.0%**
Heterogeneity: $I^2 = 0\%$ [0.0%; 89.6%], $\tau^2 = 0$, $p = 0.4015$									
**Odds Ratio**									
**Chawla et al., 2014**	Coronary artery disease	1.4	R	Death, chronic KRT, 25% eGFR decrease	70/76	9633/6558 (68%)	18921/7351 (39%)	3.36 [3.19; 3.54]	21.5%
**Chawla et al., 2017**	Infection or sepsis	1.8	R	Death, chronic KRT, 25% eGFR decrease	74/78	13390/8262 (62%)	33067/15825 (48%)	1.76 [1.68; 1.83]	21.7%
**Assess−AKI, 2020 & 2021**	Hospital	4.7	P	Death, chronic KRT or new eGFR <15, 50% eGFR decrease, new eGFR <60	67/70	769/358 (47%)	769/192 (25%)	2.62 [2.11; 3.25]	19.4%
**Glasbey, 2021**	Surgery (non−cardiovascular)	1.0	P	Death, chronic KRT, 30% eGFR decrease	−/−	475/128 (27%)	3029/250 (8%)	4.10 [3.22; 5.22]	15.8%
**See et al., 2021**	Hospital	2.6	R	Death, chronic KRT or new eGFR <15, new eGFR <60	86/91	3379/1011 (30%)	32262/5250 (16%)	2.20 [2.03; 2.38]	21.5%
**Chen, L. et al. (AKIN), 2022**	Coronary artery disease	1.0	P	Death, chronic KRT, 50% SCr increase	80/91	41/16 (39%)	361/13 (4%)	17.13 [7.42; 39.56]	0.2%
**Random effects model**								**2.77 [2.01; 3.53]**	**100.0%**
Heterogeneity: $I^2 = 98.3\%$ [97.6%; 98.8%], $\tau^2 = 0.6915$, $p < 0.0001$									

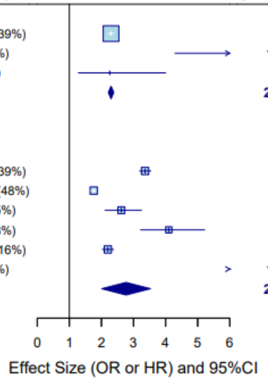

AKI, acute kidney injury; AKIN, Acute kidney injury network; CI, confidence interval; CKD chronic kidney disease; eGFR, estimated glomerular filtration rate; HR, hazard ratio; KRT, kidney replacement therapy; MAKE, major adverse kidney event; OR, odds ratio. *Outcome is adjusted for baseline kidney function.

Figure S6A. Meta-regression lines to analyse the effect of covariates on the association between acute kidney injury and CKD incidence

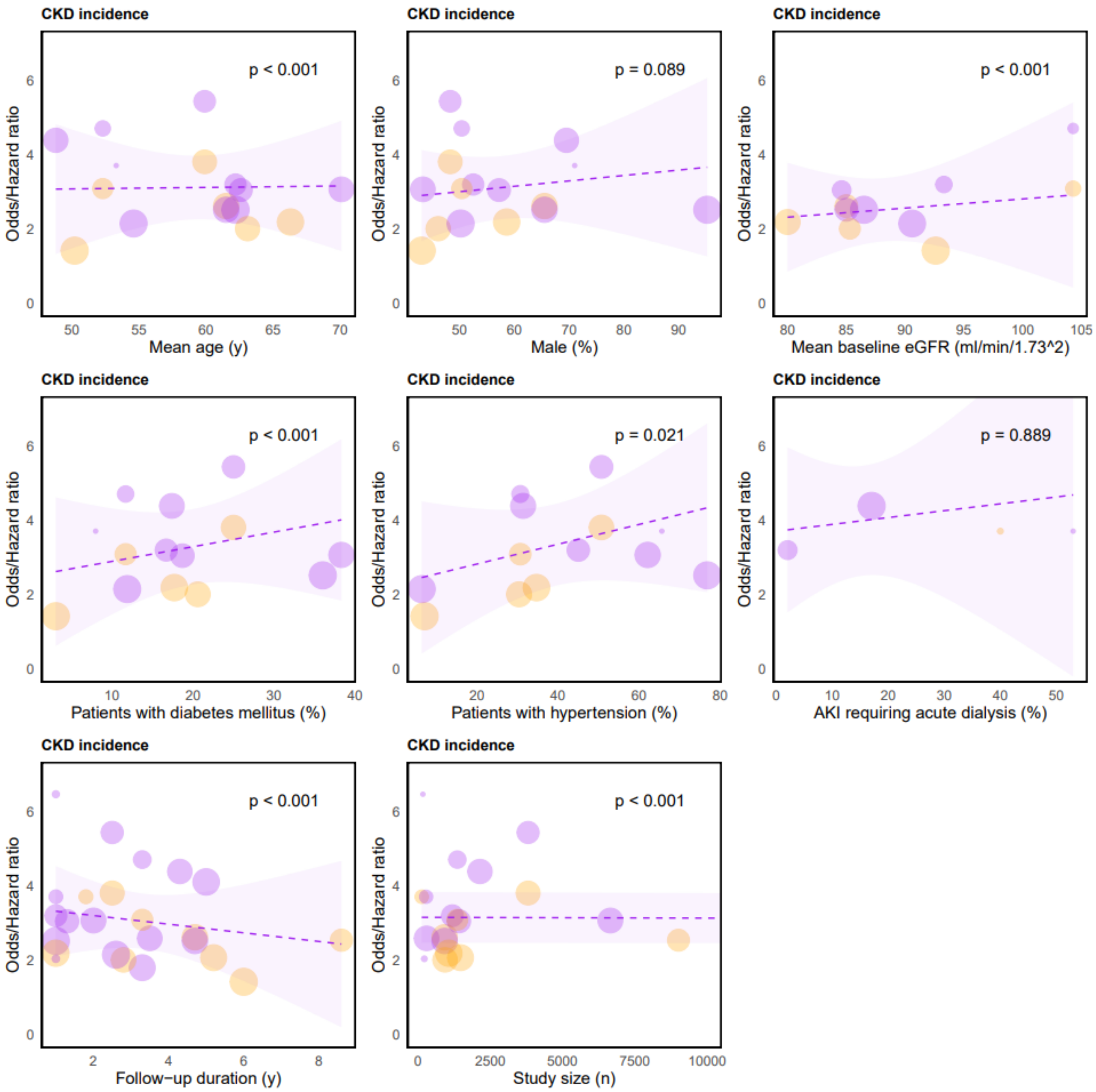

Every study is represented by a circle. Circle size indicates the study's weight in the random effects model. The line indicates the regression line with 95% confidence intervals. Purple dots represent odds ratios, orange dots represent hazard ratios. Preferably, hazard ratios were included in the meta-regression; however, if hazard ratios were not reported, odds ratios were used instead. CKD, chronic kidney disease; eGFR, estimated glomerular filtration rate; HR, hazard ratio; OR, odds ratio

Figure S6B. Meta-regression lines to analyse the effect of covariates on the association between acute kidney injury and Kidney Failure

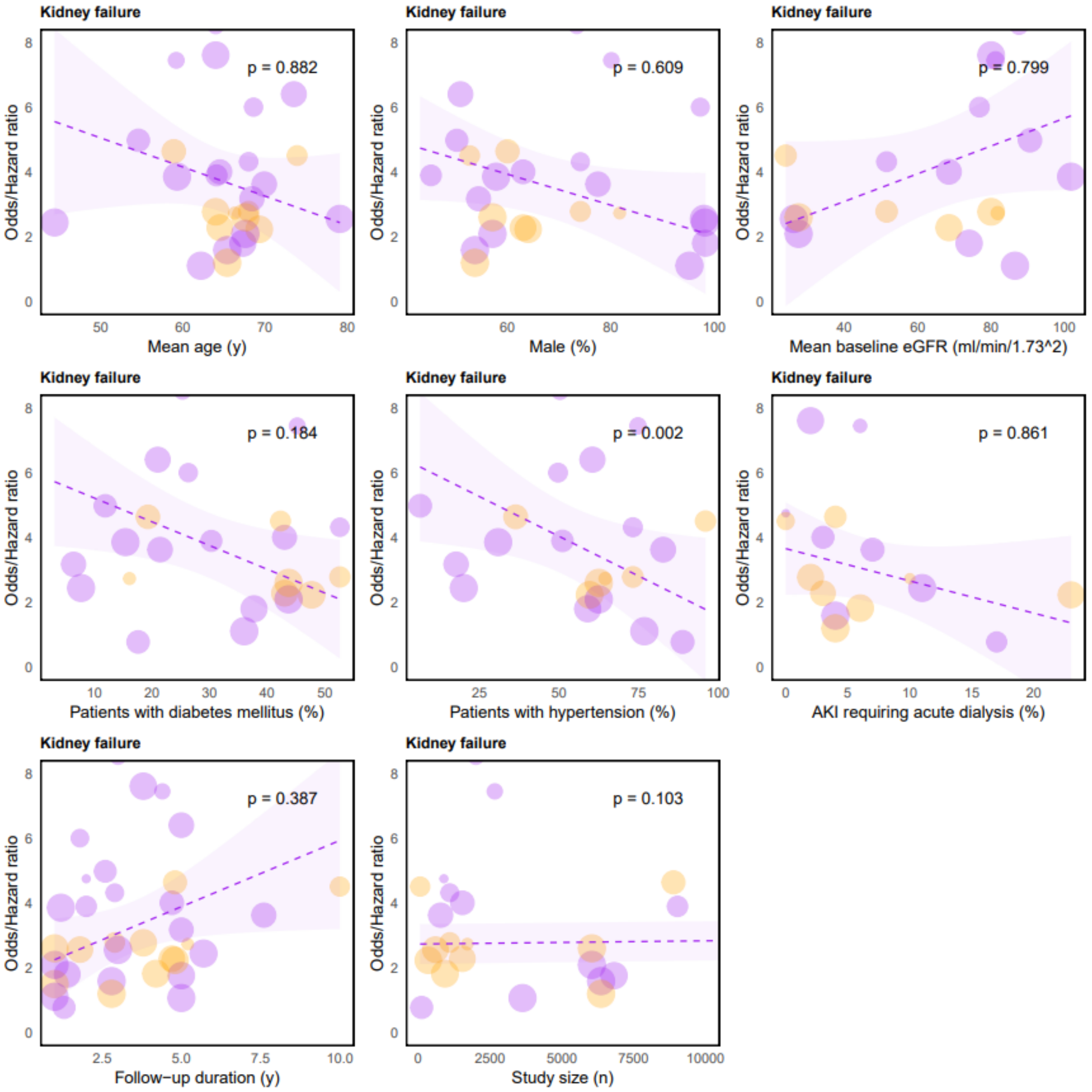

Every study is represented by a circle. Circle size indicates the study's weight in the random effects model. The line indicates the regression line with 95% confidence intervals. Purple dots represent odds ratios, orange dots represent hazard ratios. Preferably, hazard ratios were included in the meta-regression; however, if hazard ratios were not reported, odds ratios were used instead. CKD, chronic kidney disease; eGFR, estimated glomerular filtration rate; HR, hazard ratio; OR, odds ratio

Figure S6C. Meta-regression lines to analyse the effect of covariates on the association between acute kidney injury and Kidney Failure, stratified for follow-up duration

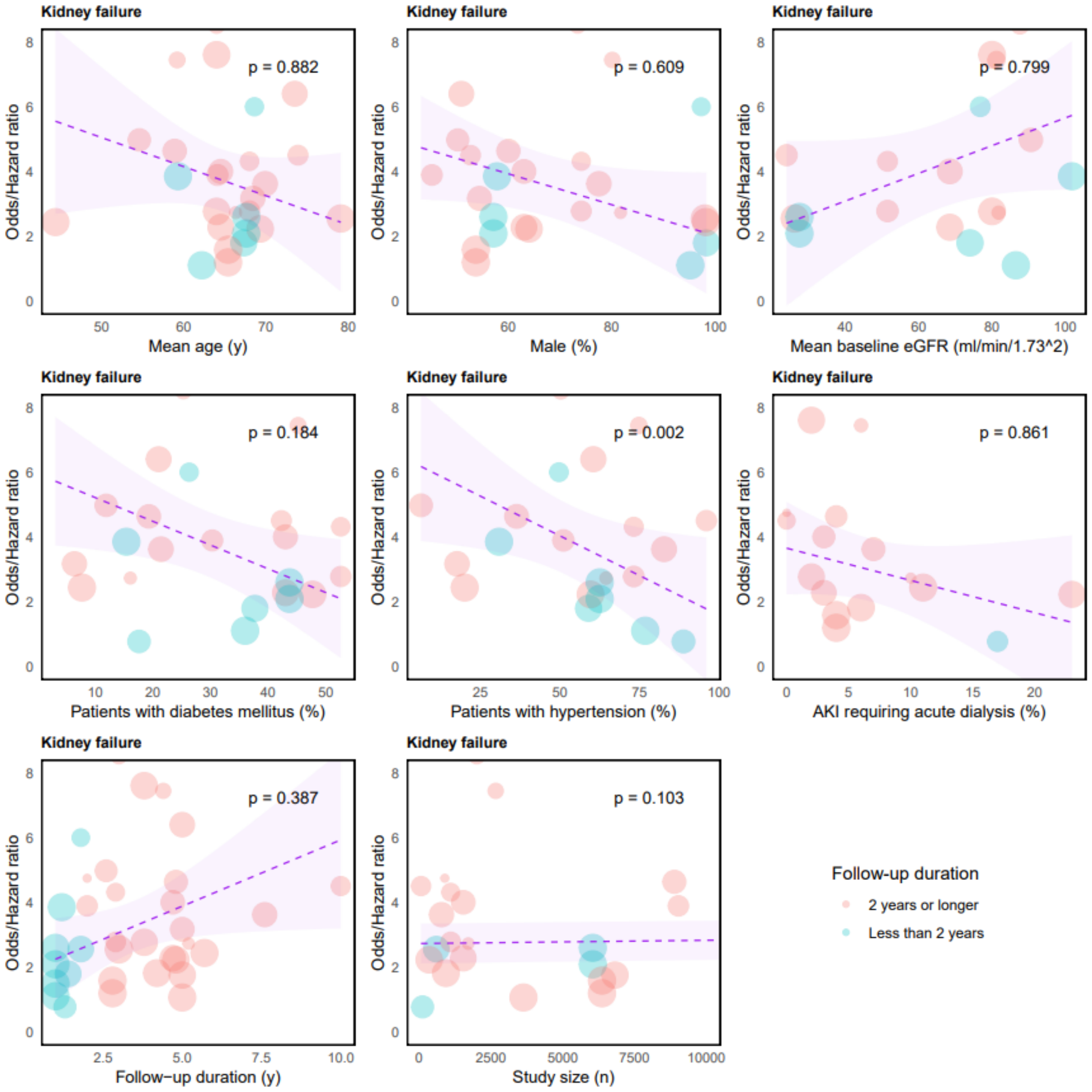

Every study is represented by a circle. Circle size indicates the study's weight in the random effects model. The line indicates the regression line with 95% confidence intervals. CKD, chronic kidney disease; eGFR, estimated glomerular filtration rate; KRT, kidney replacement therapy; HR, hazard ratio; OR, odds ratio
