# Supplementary material for: Long-term risks of adverse kidney outcomes after acute kidney injury: a systematic review and meta-analysis
Source: Nephrol Dial Transplant. 2025 May 27;40(11):2143–58. doi: 10.1093/ndt/gfaf093 (PMC12559794; doi:10.1093/ndt/gfaf093)
Supplement: gfaf093_Supplemental_Files [file gfaf093_supplemental_files.zip › C.Tables_Supplements_S1_to_S3_20022025.pdf]

Supplementary material including:

- **Table S1.** Search strategy
- **Table S2.** Reasons for exclusion in full-text assessment
- **Table S3.** Risk of bias assessment

Table S1. Search strategy

A. Search Medline through PubMed *(October 2 2024)*

#	Query		
1	(“acute kidney injury”[Mesh]	OR	
	“acute kidney injur*”[ti] OR “acute on chronic kidney injur*”[ti]	OR	
	“acute kidney failure”[ti] OR “acute on chronic kidney failure”[ti]	OR	
	“acute kidney impairment”[ti]	OR	
	“acute kidney insufficienc*”[ti]	OR	
	“acute kidney disease”[ti] OR “acute on chronic kidney disease”[ti]	OR	
	“acute renal injur*”[ti] OR “acute on chronic renal injur*”[ti]	OR	
	“acute renal failure”[ti] OR “acute on chronic renal failure”[ti]	OR	
	“acute renal impairment”[ti]	OR	
	“acute renal insufficienc*”[ti]	OR	
	“acute renal disease*”[ti] OR “acute on chronic renal disease*”[ti]	OR	
	“acute tubular necros*”[ti]	OR	
	((“surgery” [ti] OR “contrast” [ti] OR “sepsis” [ti] OR “IC”[ti] OR “intensive care” [ti]) AND (“kidney injur*”[ti] OR “renal injur*”[ti]))	OR	
	((“surgery” [ti] OR “contrast” [ti] OR “sepsis” [ti] OR “IC”[ti] OR “intensive care” [ti]) AND (“kidney failure”[ti] OR “renal failure”[ti]))	OR	
	((“surgery” [ti] OR “contrast” [ti] OR “sepsis” [ti] OR “IC”[ti] OR “intensive care” [ti]) AND (“kidney impairment”[ti] OR “renal impairment”[ti]))	OR	
	((“surgery” [ti] OR “contrast” [ti] OR “sepsis” [ti] OR “IC”[ti] OR “intensive care” [ti]) AND (“kidney insufficienc*”[ti] OR “renal insufficienc*”[ti]))	OR	
	((“surgery” [ti] OR “contrast” [ti] OR “sepsis” [ti] OR “IC”[ti] OR “intensive care” [ti]) AND (“kidney disease”[ti] OR “renal disease”[ti]))	OR	
	“AKIN“[ti] OR “RIFLE“[ti] OR (“KDIGO“[ti] AND “acute“[ti]) OR (“KDIGO“[ti] AND “AKI”[ti])	OR	
	”AKI”[ti] OR ”AKF”[ti] OR ”AKD”[ti] OR ”ARI” [ti] OR ”ARF”[ti] OR ”ARD”[ti] OR ”ATN”[ti]	)	69.023
	AND		
2	("Renal Insufficiency, Chronic"[Mesh]	OR	
	“chronic kidney disease”[tiab]	OR	
	“chronic kidney failure”[tiab]	OR	
	“chronic kidney impairment”[tiab]	OR	
	“chronic kidney insufficienc*”[tiab]	OR	
	“chronic renal disease”[tiab]	OR	
	“chronic renal failure”[tiab]	OR	
	“chronic renal impairment”[tiab]	OR	
	“chronic renal insufficienc*”[tiab]	OR	
	“end stage kidney disease”[tiab] OR “end stage renal disease”[tiab] OR “end stage renal failure”[tiab]	OR	
	“kidney failure”[tiab] OR “renal failure”[tiab]	OR	
	“CKD”[tiab] or “CKF”[tiab] or “CKI”[tiab] or “CRD”[tiab] or “CRF”[tiab] or “CRI”[tiab] or “ESRD”[tiab] or “ESRF”[tiab] or “ESKD”[tiab] or “ESKF”[tiab]	OR	
	"Cardiovascular Diseases"[Mesh]	OR	
	“cardiovascular disease*”[tiab] or “CVD”[tiab] or “heart failure”[tiab] or “myocardial infarc*”[tiab] or “MI”[tiab] or “stroke”[tiab]	OR	
	"Death"[Mesh]	OR	
	“death”[tiab]	OR	
	“mortality”[tiab]	OR	
	“survival”[tiab]	)	5.685.900
	AND		
3	(“outcome*”[tiab]	OR	
	“progression”[tiab]	OR	
	“prognosis”[tiab]	OR	
	“follow up”[tiab] OR “followup”[tiab] or “follow-up”[tiab]	)	4.394.893
	#1 AND #3		17.560
	#1 AND #2 AND #3		13.966
	limit yr="2004 - current"		12.437
	limit yr="2018 - current"		7.209

B. Search Embase through Embase.com *(October 2 2024)*

#	Query		
1	(‘acute kidney failure’/mj	OR	
	‘acute kidney injury’:ti	OR	
	‘acute kidney impairment’:ti	OR	
	‘acute kidney insufficiency’:ti	OR	
	‘acute kidney disease’:ti	OR	
	‘acute kidney failure’:ti	OR	
	‘acute renal injury’:ti	OR	
	‘acute renal failure’:ti	OR	
	‘acute renal impairment’:ti	OR	
	‘acute renal insufficiency’:ti	OR	
	‘acute renal disease’:ti	OR	
	‘Acute tubular necrosis’:ti	OR	
	‘acute-on-chronic kidney injury’:ti	OR	
	‘acute on chronic kidney injury’:ti	OR	
	((‘contrast‘:ti OR ‘surgery‘:ti OR ‘sepsis’:ti OR ‘IC’:ti OR ‘intensive care’:ti) AND (‘kidney injury‘:ti OR ‘renal injury’:ti))	OR	
	((‘contrast‘:ti OR ‘surgery‘:ti OR ‘sepsis’:ti OR ‘IC’:ti OR ‘intensive care’:ti) AND (‘kidney failure’:ti OR ‘renal failure’:ti))	OR	
	((‘contrast‘:ti OR ‘surgery‘:ti OR ‘sepsis’:ti OR ‘IC’:ti OR ‘intensive care’:ti) AND (‘kidney impairment’:ti OR ‘renal impairment’:ti))	OR	
	((‘contrast‘:ti OR ‘surgery‘:ti OR ‘sepsis’:ti OR ‘IC’:ti OR ‘intensive care’:ti) AND (‘kidney insufficiency’:ti OR ‘renal insufficiency’:ti))	OR	
	((‘contrast‘:ti OR ‘surgery‘:ti OR ‘sepsis’:ti OR ‘IC’:ti OR ‘intensive care’:ti) AND (‘kidney disease’:ti OR ‘renal disease’:ti))	OR	
	‘acute kidney tubular necrosis’:ti	OR	
	‘AKI’:ti or ‘AKF’:ti or ‘AKD’:ti or ‘ARI’:ti or ‘ARF’:ti or ‘ARD’:ti or ‘ATN’:ti	)	67.587
	AND		
2	('chronic kidney failure'/exp	OR	
	'chronic kidney impairment':ab,ti	OR	
	'chronic kidney insufficiency':ab,ti	OR	
	‘chronic renal disease':ab,ti	OR	
	'chronic renal failure':ab,ti	OR	
	'chronic renal impairment':ab,ti	OR	
	'chronic renal insufficiency':ab,ti	OR	
	'end stage renal disease'/exp	OR	
	‘end stage renal failure':ab,ti	OR	
	'end stage kidney disease':ab,ti OR 'end stage renal disease':ab,ti	OR	
	'kidney failure':ab,ti	OR	
	'ckd':ab,ti OR 'ckf':ab,ti OR 'cki':ab,ti OR 'crd':ab,ti OR 'crf':ab,ti OR 'cri':ab,ti OR 'esrd':ab,ti OR 'esrf':ab,ti OR 'eskd':ab,ti OR 'eskf':ab,ti	OR	
	'cardiovascular disease'/exp	OR	
	'cardiovascular disease':ab,ti or 'CVD':ab,ti or 'heart failure':ab,ti or 'heart infarction':ab,ti or 'myocardial infarction':ab,ti or 'MI':ab,ti or 'stroke':ab,ti	OR	
	'death'/exp	OR	
	'death':ab,ti	OR	
	'mortality':ab,ti	OR	
	'survival':ab,ti	)	9.506.376
	AND		
3	(‘follow up’:ab,ti OR ‘followup’:ab,ti OR ‘follow-up’:ab,ti	OR	
	‘progression’:ab,ti	OR	
	‘outcome’:ab,ti	OR	
	‘prognosis’:ab,ti	)	5.077.809
	#1 AND #2 AND #3		12.688
	limit yr="2004 - current"		11.869
	limit yr="2018 - current"		7.004

Table S2. Reasons for exclusion in full-text assessment

Listed if the reason for exclusion was not “wrong study population” or “wrong outcome”.

Authors	Year	Title	Reason for exclusion Final
J. Jiang et al.	2014	A long-term outcome study of acute kidney injury after cardiac surgery	**PDF not found**
M. Barbanti et al.	2014	Acute kidney injury after transcatheter aortic valve implantation with self-expanding CoreValve prosthesis: results from a large multicentre Italian research project	**PDF not found**
P. J. Der Mesropian et al.	2014	Long-term outcomes of community-acquired versus hospital-acquired acute kidney injury: A retrospective analysis	**PDF not found**
C. G. Da Silveira Santos et al.	2018	Acute kidney injury in elderly population: A prospective observational study	**Wrong control group**
A. K. Bonnecaze et al.	2018	Acute kidney injury is common with intravenous abuse of extended-release oral oxymorphone and delayed renal recovery rates are associated with increased KDIGO staging	**No control group**
J. B. Rechene et al.	2018	Acute kidney injury on intensive care units. Risk factors and mortality	**PDF not found**
Z. A. Mohammed et al.	2018	Acute kidney injury: Prevalence and outcomes in southern Indian population	**Follow-up <1y**
Y. Gorelik et al.	2018	Acute Renal Failure Following Near-Drowning	**Follow-up <1y**
Y. J. Kim et al.	2018	Analysis of the development and progression of carbon monoxide poisoning-related acute kidney injury according to the Kidney Disease Improving Global Outcomes (KDIGO) criteria	**Follow-up <3mo**
J. Q. Liu et al.	2018	Characteristics of and risk factors for death in elderly patients with acute kidney injury: a multicentre retrospective study in China	**Follow-up <3mo**
A. Meraz-Muñoz et al.	2018	Chronic kidney disease after acute kidney injury associated with intravenous colistin use in survivors of severe infections: A comparative cohort study	**Wrong control group**
R. O. Mathew et al.	2018	Community acute kidney injury is associated with short- and long-term adverse outcomes in patients admitted with acute myocardial infarction	**PDF not found**
E. M. Mezhonov et al.	2018	Contrast-Induced Acute R enal Injury After Percutaneous Coronary InterventionI in Patients With ST-Segment Elevation Myocardial Infarction	**PDF not found**
T. Fujii et al.	2018	Diagnosis, management, and prognosis of patients with acute kidney injury in Japanese intensive care units: The JAKID study	**Follow-up <3mo**
P. S. Priyamvada et al.	2018	Epidemiology and outcomes of acute kidney injury in critically ill: Experience from a tertiary care center	**Follow-up <3mo**
D. Wang et al.	2018	Epidemiology of acute kidney injury in patients with stroke: a retrospective analysis from the neurology ICU	**Follow-up <3mo**
A. S. Truche et al.	2018	ICU survival and need of renal replacement therapy with respect to AKI duration in critically ill patients	**Follow-up <3mo**
E. Rodríguez et al.	2018	Impact of Recurrent Acute Kidney Injury on Patient Outcomes	**Wrong control group**
Li et al.	2018	Incidence and short-term outcomes of acute kidney injury in very elderly patients	**Not in English**
M. P. E. Halle et al.	2018	Incidence, characteristics and prognosis of acute kidney injury in Cameroon: a prospective study at the Douala General Hospital	**Wrong control group**
S. Morton et al.	2018	Is Frailty a Predictor of Outcomes in Elderly Inpatients with Acute Kidney Injury? A Prospective Cohort Study	**Follow-up <3mo**
M. Meersch et al.	2018	Long-term clinical outcomes after early initiation of RRT in critically Ill patients with AKI	**Wrong control group**
González et al.	2018	Non-critical care hospital-acquired acute kidney injury. Analysis of 101 cases	**Not in English**
M. Eskola et al.	2018	One- and three-year outcomes in patients treated with intermittent hemodialysis for acute kidney injury: prospective observational multicenter post-hoc FINNAKI study	**Wrong control group**
J. S. Kim et al.	2018	One--year progression and risk factors for the development of chronic kidney disease in septic shock patients with acute kidney injury: A single-centre retrospective cohort study	**Wrong control group**
V. C. Wu et al.	2018	Outcome prediction of acute kidney injury biomarkers at initiation of dialysis in critical units	**Wrong control group**
E. Gabbay et al.	2018	Outcomes of very elderly treated with dialysis for acute kidney injury	**PDF not found**
S. Park et al.	2018	Postdischarge Major Adverse Cardiovascular Events of ICU Survivors Who Received Acute Renal Replacement Therapy	**Wrong control group**
K. A. Mizuguchi et al.	2018	Predicting kidney disease progression in patients with acute kidney injury after cardiac surgery	**Follow-up <3mo**
G. Geri et al.	2018	Prediction of chronic kidney disease after acute kidney injury in ICU patients: study protocol for the PREDICT multicenter prospective observational study	**Wrong study design**
S. Hanoura et al.	2018	Prevalence and predictors of acute kidney injury after cardiac surgery: A single-centre retrospective study in Qatar	**PDF not found**
A. Joseph et al.	2018	Renal recovery after severe acute kidney injury in critically ill myeloma patients: A retrospective study	**Follow-up <3mo**
L. V. Kremneva et al.	2018	Risk factors and in-hospital outcomes of acute kidney injury that developed after coronary artery bypass grafting in patients with stable angina	**Not in English**
C. Arias-Cabrales et al.	2018	Short- and long-term outcomes after non-severe acute kidney injury	**Wrong control group**
T. H. Mallhi et al.	2018	Short-term renal outcomes following acute kidney injury among dengue patients: A follow-up analysis from large prospective cohort	**Follow-up <3mo**
S. Attard et al.	2018	The incidence, predictors, and prognosis of acute kidney injury after transcatheter aortic valve implantation	**PDF not found**
S. Khoury et al.	2019	Acute renal impairment in older adults treated with percutaneous coronary intervention for ST-segment elevation myocardial infarction	**Follow-up <1y**
I. J. Pranjić et al.	2019	Chronic kidney disease after acute renal injury	**Not in English**
J. Wang et al.	2019	Clinical characteristics and prognosis of acute kidney injury in elderly patients with sepsis	**Not in English**
S. Y. Zhang et al.	2019	Clinical features and prognosis of infective endocarditis patients with acute kidney injury	**Not in English**
W. Ribitsch et al.	2019	Contrast Induced Acute Kidney Injury and its Impact on Mid-Term Kidney Function, Cardiovascular Events and Mortality	**Follow-up <1y**
J. Zhang et al.	2019	Cumulative fluid accumulation is associated with the development of acute kidney injury and non-recovery of renal function: A retrospective analysis	**Follow-up <3mo**
E. H. Lee et al.	2019	Effects on renal outcome of concomitant acute pyelonephritis, acute kidney injury and obstruction duration in obstructive uropathy by urolithiasis: a retrospective cohort study	**Follow-up <1y**
H. Wang et al.	2019	Higher body mass index is not a protective risk factor for 28-days mortality in critically ill patients with acute kidney injury undergoing continuous renal replacement therapy	**Wrong control group**
B. H. Yang et al.	2019	High-sensitivity C-reactive protein as a predictive factor of acute kidney injury following aneurysmal subarachnoid hemorrhage: a prospective observational study	**Follow-up <3mo**

McAdams et al.	2019	Kidney recovery in patients discharged to an acute rehabilitation facility with acute kidney injury requiring hemodialysis	**PDF not found**
S. W. Chen et al.	2019	Long-term outcomes after extracorporeal membrane oxygenation in patients with dialysis-requiring acute kidney injury: A cohort study	**Wrong control group**
H. L. MacLaughlin et al.	2019	Obesity and recovery from acute kidney injury (Ob AKI): A prospective cohort feasibility study	**No control group**
S. Singh et al.	2019	Outcomes of hospital-acquired acute kidney injury in elderly patients: a single-centre study	**Follow-up <3mo**
F. Husain-Syed et al.	2019	Persistent decrease of renal functional reserve in patients after cardiac surgery-associated acute kidney injury despite clinical recovery	**Follow-up <1y**
S. Lee et al.	2019	Postdischarge long-term cardiovascular outcomes of intensive care unit survivors who developed dialysis-requiring acute kidney injury after cardiac surgery	**Wrong control group**
T. E. Long et al.	2019	Postoperative Acute Kidney Injury: Focus on Renal Recovery Definitions, Kidney Disease Progression and Survival	**Wrong control group**
A. Fayed et al.	2019	Prediction of mortality and need for renal replacement therapy in patients of acute kidney injury using fibroblast growth factor 23	**PDF not found**
J. Thanavaro et al.	2019	Predictors and Outcomes of Acute Kidney Injury after Cardiac Surgery	**PDF not found**
K. Sueyoshi et al.	2019	Predictors of long-term prognosis in acute kidney injury survivors who require continuous renal replacement therapy after cardiovascular surgery	**Wrong control group**
C. Josa-Laorden et al.	2019	Prognostic significance of acute kidney injury and small increases in creatinine concentration during acute decompensation of heart failure	**Not in English**
M. Eswarappa et al.	2019	Renal manifestations of dengue viral infection	**PDF not found**
George et al.	2019	Renal outcomes among snake-envenomed patients with acute kidney injury in southern India	**PDF not found**
S. V. Avdoshina et al.	2019	Risk assessment of acute kidney injury in patients with acute cardiovascular disease without invasive intervention	**Not in English**
R. E. Aylward et al.	2019	Risk factors and outcomes of acute kidney injury in South African critically ill adults: a prospective cohort study	**Wrong control group**
Z. Y. Xie et al.	2019	Risk factors of postoperative acute kidney injury and the impact on outcome in non-senile patients undergoing cardiac valvular surgery	**Not in English**
Xie et al.	2019	Risk factors of postoperative acute kidney injury and the impact on outcome in non-senile patients undergoing cardiac valvular surgery	**PDF not found**
H. D. N. Lima et al.	2019	The impact of acute kidney injury on fatality of ischemic stroke from a hospital-based population in Joinville, Brazil	**Follow-up <3mo**
S. Rubin et al.	2019	The incidence of chronic kidney disease three years after non-severe acute kidney injury in critically ill patients: A single-center cohort study	**Wrong control group**
L. Sykes et al.	2019	The influence of multiple episodes of acute kidney injury on survival and progression to end stage kidney disease in patients with chronic kidney disease	**Wrong control group**
M. Xie et al.	2020	Acute kidney injury diagnosed by elevated serum creatinine increases mortality in ICU patients following non-cardiac surgery	**Not in English**
P. T. Hanna et al.	2020	Acute Kidney Injury following Enhanced Recovery after Surgery in Patients Undergoing Radical Cystectomy	**Follow-up <1y**
S. P. Jacups et al.	2020	Acute kidney injury in Indigenous intensive care patients	**No control group**
J. Chen et al.	2020	Clinical characteristics of infection-induced acute renal injury in acute-on-chronic liver failure	**Not in English**
Pampa-Saico et al.	2020	Colistimethate sodium and acute kidney injury: Incidence, risk factors, outcome and prognosis of renal function	**Follow-up <1y**
S. D. Weisbord et al.	2020	Contrast-Associated Acute Kidney Injury and Serious Adverse Outcomes Following Angiography	**Follow-up <1y**
C. Thongprayoon et al.	2020	Diagnostics, risk factors, treatment and outcomes of acute kidney injury in a new paradigm	**Wrong study design**
C. Liu et al.	2020	Drug-induced acute kidney injury in hospitalized patients: a retrospective study	**PDF not found**
Kee et al.	2020	Dyschloremia is associated with failure to restore renal function in survivors with acute kidney injury: an observation retrospective study	**Wrong control group**
H. R. Chua et al.	2020	Extended Mortality and Chronic Kidney Disease After Septic Acute Kidney Injury	**Wrong control group**
M. K. Khoury et al.	2020	Fenestrated-branched endovascular aortic repair in patients with chronic kidney disease	**Wrong control group**
C. Carlino et al.	2020	First epidemiological study of acute kidney injury with requirement of renal replacement therapy in Argentina. Intermediate general and renal survival	**PDF not found**
H. Iram et al.	2020	Frequency of Risk Factors and Outcome of Hospital-Acquired Acute Kidney Injury	**No control group**
Q. Li et al.	2020	Hospital-acquired acute kidney injury in very elderly men: clinical characteristics and short-term outcomes	**Follow-up <3mo**
S. Büttner et al.	2020	Incidence, Risk Factors, and Outcome of Acute Kidney Injury in Neurocritical Care	**Follow-up <3mo**
C. K. Chan et al.	2020	Long-term outcomes following vehicle trauma related acute kidney injury requiring renal replacement therapy: a nationwide population study	**Wrong control group**
T. Chen et al.	2020	Long-term predictive value of acute kidney injury classification in diffuse proliferative lupus nephritis with acute kidney injury	**Wrong control group**
H. Kim et al.	2020	Long-term Renal Outcome of Biopsy-proven Acute Tubular Necrosis and Acute Interstitial Nephritis	**Wrong control group**
W. Cheng et al.	2020	Post-contrast acute kidney injury in a hospitalized population: short-, mid-, and long-term outcome and risk factors for adverse events	**Wrong control group**
M. S. Abdalrahim et al.	2020	Pre-existing chronic kidney disease and acute kidney injury among critically ill patients	**Follow-up <3mo**
P. S. Priyamvada et al.	2020	Prognosis and long-term outcomes of acute kidney injury due to snake envenomation	**No control group**
A. Göçken et al.	2020	Retrospective evaluation of the factors affecting etiology and prognosis of adult acute kidney injury	**Not in English**
W. Zhang et al.	2020	Risk factors of acute kidney injury in hospitalized patients with infective endocarditis and their predictive values	**Not in English**
P. N. H. Tuan et al.	2020	Serum and Urine Neutrophil Gelatinase-Associated Lipocalin Levels Measured at Admission Predict Progression to Chronic Kidney Disease in Sepsis-Associated Acute Kidney Injury Patients	**Wrong control group**
Y. F. Zou et al.	2020	Serum pre-albumin is prognostic for all-cause mortality in patients with community-acquired and post-operative acute kidney injury	**Wrong control group**
R. Wang et al.	2020	Serum Procalcitonin Level Predicts Acute Kidney Injury After Traumatic Brain Injury	**Follow-up <3mo**
S. González Sanchidrián et al.	2020	Survival and renal recovery after acute kidney injury requiring dialysis outside of intensive care units	**No control group**
E. D. Siew et al.	2020	Timing of Recovery From Moderate to Severe AKI and the Risk for Future Loss of Kidney Function	**Wrong control group**
O. Adegbala et al.	2020	Trends, Outcomes, and Readmissions Among Left Ventricular Assist Device Recipients with Acute Kidney Injury Requiring Hemodialysis	**PDF not found**
R. Wiersema et al.	2020	Two subphenotypes of septic acute kidney injury are associated with different 90-day mortality and renal recovery	**Wrong control group**
I. Merdler et al.	2021	Acute cardiorenal anemia syndrome among ST-elevation myocardial infarction patients treated by primary percutaneous intervention	**PDF not found**
J. Gameiro et al.	2021	Acute kidney disease and long-term outcomes in critically ill acute kidney injury patients with sepsis: a cohort analysis	**Wrong control group**
P. Yan et al.	2021	Acute kidney disease in hospitalized acute kidney injury patients	**Wrong control group**
T. S. Kister et al.	2021	Acute kidney injury and its progression in hospitalized patients-Results from a retrospective multicentre cohort study with a digital decision support system	**Follow-up <3mo**
E. Katsogridakis et al.	2021	Acute kidney injury following endovascular intervention for peripheral artery disease	**Follow-up <1y**

A. Saverymuthu et al.	2021	Acute Kidney Injury following Rhabdomyolysis in Critically Ill Patients	**Follow-up <3mo**
T. Chen et al.	2021	Acute kidney injury in idiopathic membranous nephropathy with nephrotic syndrome	**Follow-up <1y**
A. Haase-Fielitz et al.	2021	Acute kidney injury may impede results after transcatheter aortic valve implantation	**Follow-up <1y**
S. Pillai et al.	2021	Acute Kidney Injury Post-Percutaneous Nephrolithotomy (PNL): Prospective Outcomes from a University Teaching Hospital	**No control group**
L. Dagneaux et al.	2021	Acute Kidney Injury When Treating Periprosthetic Joint Infections after Total Knee Arthroplasties with Antibiotic-Loaded Spacers: Incidence, Risks, and Outcomes	**No control group**
K. Asmus et al.	2021	AKI Epidemiology and Outcomes: A Retrospective Cohort Study from the Prenephrology Era	**Follow-up <3mo**
D. Luu et al.	2021	Association of Severe Acute Kidney Injury with Mortality and Healthcare Utilization Following Isolated Traumatic Brain Injury	**Follow-up <3mo**
K. Ariga et al.	2021	Chronic Kidney Disease after Snake Envenomation Induced Acute Kidney Injury	**No control group**
S. S. E. Khayat et al.	2021	Epidemiological Study of Acute Kidney Injury in Intensive Care Unit: Evolution and Prognosis	**No control group**
C. Salathé et al.	2021	Epidemiology and outcomes of elderly patients requiring renal replacement therapy in the intensive care unit: an observational study	**No control group**
C. W. Yang et al.	2021	Epidemiology and the Impact of Acute Kidney Injury on Outcomes in Patients with Rhabdomyolysis	**Follow-up <3mo**
E. C. Bjornstad et al.	2021	High risk of acute kidney injury in Malawian trauma patients: a prospective observational cohort study	**Follow-up <1y**
J. Yang et al.	2021	Impact of acute kidney injury on long-term adverse outcomes in obstructive uropathy	**Wrong control group**
L. Tan et al.	2021	Impact of diabetes mellitus on short-term prognosis, length of stay, and costs in patients with acute kidney injury: A nationwide survey in China	**Follow-up <3mo**
R. Wang et al.	2021	Incidence and Burden of Acute Kidney Injury among Traumatic Brain-Injury Patients	**Follow-up <3mo**
C. H. Chang et al.	2021	Incidence and Transition of Acute Kidney Injury, Acute Kidney Disease to Chronic Kidney Disease after Acute Type A Aortic Dissection Surgery	**Wrong control group**
M. J. Lommen et al.	2021	Incidence of Acute and Chronic Renal Failure Following Branched Endovascular Repair of Complex Aortic Aneurysms	**No control group**
Ch; J. rasekhar et al.	2021	Incidence, predictors, and outcomes associated with acute kidney injury in patients undergoing transcatheter aortic valve replacement: from the BRAVO-3 randomized trial	**Follow-up <3mo**
J. H. Yoon et al.	2021	Is acute kidney injury after laparoscopic adrenalectomy related to the progression of chronic kidney disease in patients with primary aldosteronism?	**Follow-up <1y**
Weidemann et al.	2021	Long-term clinical outcomes of acute kidney failure	**Not in English**
L. Mizera et al.	2021	Long-term outcome after dialysis-dependent renal failure on the intensive care unit	**Not in English**
J. J. Chen et al.	2021	Long-term outcomes of acute kidney injury after different types of cardiac surgeries: A population-based study	**Wrong control group**
F. Mariano et al.	2021	Long-term preservation of renal function in septic shock burn patients requiring renal replacement therapy for acute kidney injury	**Wrong control group**
G. Vasquez-Rios et al.	2021	Molecular and clinical signatures in Acute Kidney Injury define distinct subphenotypes that associate with death, kidney, and cardiovascular events	**Wrong control group**
Zika et al.	2021	Older People and Acute Kidney Injury: A Student Perspective on Medication Changes During Hospital Admission and Transitions of Care Follow-Up	**PDF not found**
T. K. Oh et al.	2021	Postoperative acute kidney injury requiring continuous renal replacement therapy and outcomes after coronary artery bypass grafting: a nationwide cohort study	**Wrong control group**
A. Arshad et al.	2021	Progression of Acute Kidney Injury to Chronic Kidney Disease in Sepsis Survivors: 1-Year Follow-Up Study	**No control group**
Brar et al.	2021	Prospective cohort study of renin-angiotensin system blocker usage after hospitalized acute kidney injury	**Wrong control group**
E. Macedo et al.	2021	Recognition and management of community-acquired acute kidney injury in low-resource settings in the ISN 0by25 trial: A multi-country feasibility study	**Follow-up <1y**
E. J. See et al.	2021	Risk factors for major adverse kidney events in the first year after acute kidney injury	**Wrong control group**
A. H. Flannery et al.	2021	Sepsis-Associated Acute Kidney Disease and Long-term Kidney Outcomes	**Follow-up <1y**
W. Wei et al.	2021	Short-term prognosis and influencing factors of patients with acute kidney injury treated with prolonged intermittent renal replacement therapy	**Follow-up <3mo**
Y. Wen et al.	2021	The aftermath of AKI: Recurrent AKI, acute kidney disease, and CKD progression	**Wrong study design**
P. Priyanka et al.	2021	The impact of acute kidney injury by serum creatinine or urine output criteria on major adverse kidney events in cardiac surgery patients	**Follow-up <1y**
M. Mortazavi et al.	2021	The incidence rate of acute kidney injury and risk factors among the intensive care unit inpatients	**PDF not found**
W. Hu et al.	2021	The incidence, characteristics, and use of suspected nephrotoxic drugs in elderly patients with community-acquired acute kidney injury	**Follow-up <3mo**
S. Hapca et al.	2021	The relationship between AKI and CKD in patients with type 2 diabetes: An observational cohort study	**Wrong control group**
T. Avni et al.	2021	The significance of acute kidney injury in Clostridioides difficile infection	**Follow-up <1y**
G. Nascimento et al.	2021	Type 1 Cardiorenal Syndrome in Decompensated Heart Failure Patients in a Low-Income Region in Brazil: Incidence of Acute Kidney Injury (AKIN and KDIGO Criteria), Need for Dialysis and Mortality	**Not in English**
J. G. Amatruda et al.	2021	Urine Alpha-1-Microglobulin Levels and Acute Kidney Injury, Mortality, and Cardiovascular Events following Cardiac Surgery	**Wrong control group**
L. V. Kremneva et al.	2022	Acute Kidney Injury after Transcatheter Aortic Valve Implantation	**Not in English**
R. Vairakkani et al.	2022	Acute kidney injury in a tertiary care center of South India	**Follow-up <1y**
Demchuk et al.	2022	ACUTE KIDNEY INJURY IN PATIENTS WITH MYOCARDIAL INFARCTION AND PERCUTANEOUS CORONARY INTERVENTIONS: LABORATORY MARKERS AND THE INFLUENCE ON THE FREQUENCY OF RECURRENT CARDIOVASCULAR EVENTS	**Not in English**
Z. Y. Huang et al.	2022	Acute kidney injury in traumatic brain injury intensive care unit patients	**Follow-up <3mo**
M. Gursu et al.	2022	Acute kidney injury in Turkey: epidemiological characteristics, etiology, clinical course, and prognosis	**No control group**
S. D. Pande et al.	2022	Acute kidney injury without need for dialysis, incidence, its impact on long-term stroke survival and progression to chronic kidney disease	**No control group**
N. M. Chadwick et al.	2022	Acute Kidney Injury: Incidence, aetiology, management and outcome measures of a Samoan case series	**Follow-up <1y**
S. G. Mansour et al.	2022	Angiopoietins as Prognostic Markers for Future Kidney Disease and Heart Failure Events After Acute Kidney Injury	**Wrong control group**
M. Wilson et al.	2022	Biomarkers During Recovery From AKI and Prediction of Long-term Reductions in Estimated GFR	**Wrong control group**
R. S. Shemiesa et al.	2022	Characteristics, risk factors and outcomes of community-acquired acute kidney injury in the elderly: a prospective tertiary hospital study, Egypt	**Wrong control group**
S. Wang et al.	2022	Clinical features and prognostic factors of acute kidney injury caused by adult secondary hemophagocytic lymphohistiocytosis	**Follow-up <3mo**
J. L. Koyner et al.	2022	Clinical Outcomes of Persistent Severe Acute Kidney Injury among Patients with Kidney Disease Improving Global Outcomes Stage 2 or 3 Acute Kidney Injury	**Follow-up <3mo**
G. Jiang et al.	2022	Clinical Predictors and Long-term Impact of Acute Kidney Injury on Progression of Diabetic Kidney Disease in Chinese Patients With Type 2 Diabetes	**Wrong study design**

F. G. Khan et al.	2022	Community acquired versus hospital acquired acute kidney injury; causes and outcome	**Wrong control group**
K. R. P. Medina et al.	2022	Comparison of Outcomes of Mild and Severe Community- and Hospital-Acquired Acute Kidney Injury	**Wrong control group**
Q. Li et al.	2022	Duration of acute kidney injury predicts 90-day mortality and chronic kidney disease progression in elderly patients	**Follow-up <3mo**
X. Luo et al.	2022	Early recovery status and outcomes after sepsis-associated acute kidney injury in critically ill patients	**Not in English**
C. Dong et al.	2022	Effect of admission mode of septic patients in intensive care unit on acute kidney injury and prognosis	**Not in English**
A. Jaryal et al.	2022	Epidemiology and outcomes of dialysis requiring acute kidney injury: A single-center study	**No control group**
A. Efat et al.	2022	Impact of indirect bilirubin and uric acid on outcomes of sepsis-associated acute kidney injury (sAKI)	**Wrong control group**
E. C. Ryan et al.	2022	Incidence and Recovery of Acute Kidney Injury in Diabetic and Nondiabetic Patients with Foot Infections	**PDF not found**
Y. L. Tain et al.	2022	Kidney Function Trajectory within Six Months after Acute Kidney Injury Inpatient Care and Subsequent Adverse Kidney Outcomes: A Retrospective Cohort Study	**Follow-up <1y**
E. Katsogridakis et al.	2022	Long-Term Effects of Acute Kidney Injury Following Endovascular Femoropopliteal Intervention: Insights From a Multicenter Trial	**Follow-up <1y**
Lim et al.	2022	Long-term renal outcome post-multimodal computed tomography in stroke evaluation	**Follow-up <1y**
D. J. Schreier et al.	2022	Nephrotoxin Exposure in the 3 Years following Hospital Discharge Predicts Development or Worsening of Chronic Kidney Disease among Acute Kidney Injury Survivors	**Wrong control group**
K. H. B. Teo et al.	2022	Neutrophil gelatinase-associated lipocalin: a biochemical marker for acute kidney injury and long-term outcomes in patients presenting to the emergency department	**Wrong control group**
H. Wang et al.	2022	Patient outcomes following AKI and AKD: a population-based cohort study	**Wrong control group**
M. K. Sahu et al.	2022	Postoperative Fluid Therapy in Adult Cardiac Surgical Patients and Acute Kidney Injury: A Prospective Observational Study	**Follow-up <1y**
E. Soum et al.	2022	Predictive factors for severe long-term chronic kidney disease after acute kidney injury requiring renal replacement therapy in critically ill patients: an ancillary study of the ELVIS randomized controlled trial	**Wrong control group**
P. C. Hsu et al.	2022	Predictors of Acute Kidney Disease Severity in Hospitalized Patients with Acute Kidney Injury	**Follow-up <1y**
Z. Chen et al.	2022	Prognostic analysis of crescentic glomerulonephritis with acute kidney injury: a single-center cohort with 5-year follow-up	**Wrong control group**
P. Esposito et al.	2022	Renal Outcomes of Dialysis-Dependent Acute Kidney Injury in Noncritically Ill Patients: A Retrospective Study	**Follow-up <1y**
D. C. Zhu et al.	2022	Rhabdomyolysis-associated acute kidney injury: clinical characteristics and intensive care unit transfer analysis	**Follow-up <3mo**
L. V. Kremneva et al.	2022	Risk Factors and Hospital Outcomes of Acute Kidney Injury in Patients Operated on for an acquired Valvular Heart Disease	**Not in English**
K. Choi et al.	2022	Risk factors for end-stage renal disease in patients with trauma and stage 3 acute kidney injury	**Wrong control group**
Y. W. Chen et al.	2022	Severe acute kidney disease is associated with worse kidney outcome among acute kidney injury patients	**Wrong control group**
M. Andonovic et al.	2022	Short- and long-term outcomes of intensive care patients with acute kidney disease	**Wrong control group**
M. Marcello et al.	2022	Subclinical AKI and Clinical Outcomes in Elderly Patients Undergoing Cardiac Surgery: Diagnostic Utility of NGAL versus Standard Creatinine Increase Criteria	**Follow-up <1y**
Li et al.	2022	The Correlation Between the Types of Initial Bacterial Infection and Clinical Prognosis in Patients With Septic AKI	**Follow-up <3mo**
L. Hu et al.	2022	The incidence, risk factors and outcomes of acute kidney injury in critically ill patients undergoing emergency surgery: a prospective observational study	**Follow-up <3mo**
Fu et al.	2022	THE RELATIONSHIP BETWEEN THE RISK FACTORS OF CI-AKI AND MACE AND THE CYS C LEVEL IN ELDERLY PATIENTS AFTER PCI	**Wrong control group**
J. A. Barea-Mendoza et al.	2022	Traumatic Brain Injury and Acute Kidney Injury-Outcomes and Associated Risk Factors	**Follow-up <3mo**
Colbert et al.	2022	Update and review of contrast-associated acute kidney injury	**Wrong study design**
Toro et al.	2023	A Combined Biomarker That Includes Plasma Fibroblast Growth Factor 23, Erythropoietin, and Klotho Predicts Short- and Long-Term Morbimortality and Development of Chronic Kidney Disease in Critical Care Patients with Sepsis: A Prospective Cohort	**Wrong control group**
Chen et al.	2023	A novel predictive model for poor in-hospital outcomes in patients with acute kidney injury after cardiac surgery	**Follow-up <3mo**
Krishna et al.	2023	A PROSPECTIVE STUDY OF EPIDEMIOLOGY, CAUSES AND PROGNOSIS OF ACUTE RENAL FAILURE IN A TERTIARY CARE HOSPITAL	**PDF not found**
Goh et al.	2023	A prospective study of incidence and outcome of acute kidney injury among hospitalised patients in Malaysia (My-AKI)	**No control group**
Kumari et al.	2023	A STUDY ON ETIOLOGY AND CLINICAL OUTCOME OF TROPICAL ACUTE KIDNEY INJURY (AKI) IN A TERTIARY CARE HOSPITAL	**PDF not found**
Rahul, A. and Kumar, S.	2023	A Tertiary Hospital Based Study of the Clinical Profile, Outcome, and Prognostic Factors of Acute Kidney Injury	**PDF not found**
S. C. V. S. Mutyala et al.	2023	Acute Kidney Injury among Post Cardiac Surgery Patients: A Retrospective Study	**PDF not found**
Kremneva et al.	2023	Acute kidney injury as a risk factor for atrial fibrillation after coronary artery bypass grafting — effects of sodium-glucose cotransporter-2 inhibitors	**Not in English**
Hamzic-Mehmedbasic et al.	2023	Acute Kidney Injury Classifications in the Prediction of In-hospital Mortality and Renal Function Non-recovery	**Follow-up <3mo**
Lake et al.	2023	An atlas of healthy and injured cell states and niches in the human kidney	**Wrong study design**
Centor et al.	2023	Annals On Call - Acute Kidney Injury and Chronic Kidney Disease Progression	**PDF not found**
Li et al.	2023	Association between furosemide administration and clinical outcomes in patients with sepsis-associated acute kidney injury receiving renal replacement therapy: a retrospective observational cohort study based on MIMIC-IV database	**Follow-up <3mo**
D. Patschan et al.	2023	Biomarker-based prediction of survival and recovery of kidney function in acute kidney injury	**Wrong study design**
C. C. Lee et al.	2023	Characteristics of and Outcomes After Dialysis-Treated Acute Kidney Injury, 2009-2018: A Taiwanese Multicenter Study	**Follow-up <3mo**
Adiyeke et al.	2023	Clinical courses of acute kidney injury in hospitalized patients: a multistate analysis	**Follow-up <3mo**
Gayathri et al.	2023	Clinical spectrum and outcomes of acute kidney injury: A prospective study from an intensive care unit of South India	**PDF not found**
A. Orieux et al.	2023	Clinical trajectories and impact of acute kidney disease after acute kidney injury in the intensive care unit: a 5-year single-centre cohort study	**Wrong control group**
Patil et al.	2023	COMMUNITY ACQUIRED ACUTE KIDNEY INJURY: A PROSPECTIVE OBSERVATIONAL STUDY	**PDF not found**
Zhao et al.	2023	Construction of anomogram for predicting the prognosis of patients with sepsis-associated acute kidney injury	**PDF not found**
Vemireddy et al.	2023	Contrast-Associated Acute Kidney Injury: Definitions, Epidemiology, Pathophysiology, and Implications	**Wrong study design**
Amico et al.	2023	Discovering predictive temporal patterns for Acute Kidney Injury from critical care data	**Wrong study design**
Mekonnen et al.	2023	Effect of Acute Kidney Injury on In-hospital Mortality in Non-critical Medical Patients in a Sub-Saharan African Country	**Follow-up <3mo**
Su et al.	2023	Epidemiology and outcomes of post-AKI proteinuria	**Wrong control group**
Nazar et al.	2023	Evaluate the Clinical Profile and Outcome of Patients of Acute Kidney Injury Due to Hemotoxic Snake Bite Envenomation	**PDF not found**
Gao et al.	2023	Extracorporeal membrane oxygenation and acute kidney injury: a single-center retrospective cohort	**Follow-up <3mo**

Gómez et al.	2023	Feasibility Assessment of a Biomarker-Guided Kidney-Sparing Sepsis Bundle: The Limiting Acute Kidney Injury Progression In Sepsis Trial	**Follow-up <3mo**
Molina Andújar et al.	2023	Impact of cardiac surgery associated acute kidney injury on 1-year major adverse kidney events	**Follow-up <1y**
A. Hino et al.	2023	Impact of cisplatin-induced acute kidney injury on long-term renal function in patients with solid tumors	**Follow-up <1y**
Lemma et al.	2023	Incidence of CKD and Death among Reproductive Age Women with Dialysis Requiring Acute Kidney Injury in Ethiopia: The Role of Obstetric Risk Factors	**Follow-up <3mo**
Khandy et al.	2023	Incidence, Determinants, and Outcome of Contrast-induced Acute Kidney Injury following Percutaneous Coronary Intervention at a Tertiary Care Hospital	**Follow-up <3mo**
P. K. Bhatraju et al.	2023	Integrated Analysis of Blood and Urine Biomarkers to Identify Acute Kidney Injury Subphenotypes and Associations With Long-term Outcomes	**Wrong control group**
Xu et al.	2023	Mortality and Cumulative Kidney Score are Associated with Transient and Persistent Acute Kidney Injury in Septic Patients: A Retrospective Study Based on MIMIC-IV	**PDF not found**
W. Xia; F. Yi; Q. Wang	2023	Mortality and differential predictive factors of transient and persistent sepsis-associated acute kidney injury	**PDF not found**
Aklilu et al.	2023	Outcomes Associated with Sodium-Glucose Cotransporter-2 Inhibitor Use in Acute Heart Failure Hospitalizations Complicated by AKI	**Follow-up <3mo**
Sousa et al.	2023	Post-renal acute kidney injury in patients with cancer: Clinical presentation and kidney and patient outcomes	**Follow-up <1y**
J. A. Neyra et al.	2023	Prediction of Mortality and Major Adverse Kidney Events in Critically Ill Patients With Acute Kidney Injury	**Wrong control group**
Shin et al.	2023	Prediction of renal recovery following sepsis-associated acute kidney injury requiring renal replacement therapy using contrast-enhanced ultrasonography	**Follow-up <3mo**
Zhang et al.	2023	Predictive value of pulse infusion index in the short-term prognosis of patients with sepsis-induced acute kidney injury	**PDF not found**
Loh et al.	2023	Preoperative shock index in major abdominal emergency surgery	**PDF not found**
C. Gross et al.	2023	Prognosis and mortality within 90 days in community-acquired acute kidney injury in the Southwest of Sweden	**Follow-up <1y**
Chaikijurajai et al.	2023	Prognostic Value of Natriuretic Peptide Levels for Adverse Renal Outcomes in Patients With Moderate to Severe Acute Kidney Injury With or Without Heart Failure	**Follow-up <3mo**
Karajizadeh et al.	2023	Risk Factors and Outcomes of Acute Kidney Injury in Trauma Patients Admitted to Critical Care Units	**PDF not found**
Ohlmeier et al.	2023	Risk of chronic kidney disease in patients with acute kidney injury following a major surgery: a US claims database analysis	**Wrong control group**
K. C. White et al.	2023	Sepsis-associated acute kidney injury in the intensive care unit: incidence, patient characteristics, timing, trajectory, treatment, and associated outcomes. A multicenter, observational study	**Follow-up <3mo**
R. Wang et al.	2023	Serum cystatin C is correlated with mortality of traumatic brain injury patients partially mediated by acute kidney injury	**Follow-up <3mo**
Wei et al.	2023	Short-term prognostic models for severe acute kidney injury patients receiving prolonged intermittent renal replacement therapy based on machine learning	**Follow-up <3mo**
Arul et al.	2023	Study On Profile Of Snake Bite Induced Acute Kidney Injury And Outcome	**PDF not found**
M. Wang et al.	2023	The effects of timing onset and progression of AKI on the clinical outcomes in AKI patients with sepsis: a prospective multicenter cohort study	**Follow-up <3mo**
Nguyen Duy et al.	2023	The Ratio of Contrast Volume/Glomerular Filtration Rate and Urine NGAL Predicts the Progression of Acute Kidney Injury to Chronic Kidney Disease in Patients After Planned Percutaneous Coronary Intervention	**Wrong control group**
Kusirisin	2023	The Role of Erythropoietin Levels in Predicting Long-Term Outcomes following Severe Acute Kidney Injury	**Wrong control group**
Jin et al.	2023	The roles of interleukin-17A in risk stratification and prognosis of patients with sepsis-associated acute kidney injury	**Follow-up <3mo**
Nishimoto et al.	2023	The use of anti-adrenergic agents as a predictor of acute kidney injury and delayed recovery of kidney function: the NARA-AKI cohort study	**PDF not found**
Kim et al.	2023	Tracking longitudinal biomarkers in burn patients with sepsis and acute kidney injury: an unsupervised clustering approach	**Follow-up <3mo**
Cheng et al.	2023	Urinary cell cycle biomarkers for the prediction of renal non-recovery in patients with septic acute kidney injury: a prospective study	**Follow-up <3mo**
C. Ng et al.	2023	Utility of frailty as a predictor of acute kidney injury in patients with aneurysmal subarachnoid hemorrhage	**Follow-up <3mo**
Xie et al.	2023	Weekend effect on the incidence and outcomes of cardiac surgery associated - acute kidney injury	**Follow-up <3mo**
Shah et al.	2024	A clinical score to predict recovery in end-stage kidney disease due to acute kidney injury	**Wrong control group**
Gao et al.	2024	A multicenter clinical study of critically ill patients with sepsis complicated with acute kidney injury in Beijing: incidence, clinical characteristics and outcomes	**PDF not found**
Kaur et al.	2024	A pilot study on the differential urine proteomic profile of subjects with community-acquired acute kidney injury who recover versus those who do not recover completely at 4 months after hospital discharge	**Wrong control group**
Liu et al.	2024	A two-tier feature selection method for predicting mortality risk in ICU patients with acute kidney injury	**Follow-up <3mo**
Meersch et al.	2024	Acute kidney disease beyond day 7 after major surgery: a secondary analysis of the EPIS-AKI trial	**Follow-up <1y**
Li et al.	2024	Acute kidney injury and cardiogenic shock severity for mortality risk stratification in patients supported with VA ECMO	**Follow-up <3mo**
Lee et al.	2024	Acute kidney injury as a prognostic marker in severe fever with thrombocytopenia syndrome	**Follow-up <3mo**
Kim et al.	2024	Acute kidney injury as a prognostic predictor of in-hospital mortality and neurological outcomes in patients after extracorporeal cardiopulmonary resuscitation	**Follow-up <3mo**
Jensen et al.	2024	Acute Kidney Injury Duration and 20-Year Risks of CKD and Cardiovascular Disease	**Wrong control group**
Butala et al.	2024	Acute Kidney Injury Following Transcatheter Aortic Valve Implantation-A Contemporary Perspective of Incidence, Predictors, and Outcomes	**Follow-up <1y**
Arfeen et al.	2024	ACUTE KIDNEY INJURY IN POSTPARTUM PATIENTS: RISK FACTORS AND OUTCOME	**PDF not found**
Buis et al.	2024	Acute kidney injury in Staphylococcus aureus bacteraemia: a recurrent events analysis	**Follow-up <1y**
Wajid et al.	2024	Acute Kidney Injury in Systemic Sclerosis Beyond Scleroderma Renal Crisis: A Case-Control Study	**Follow-up <3mo**
Babickova et al.	2024	Adverse effects of acute tubular injury on the glomerulus: contributing factors and mechanisms	**Wrong study design**
Wang et al.	2024	Association between onset time of sepsis-associated acute kidney injury and clinical outcome in patients with sepsis	**PDF not found**
Cheng et al.	2024	Association between the levels of urinary cell cycle biomarkers and non-recovery of renal function among critically ill geriatric patients with acute kidney injury	**Follow-up <3mo**
Wang et al.	2024	Attributable mortality of acute kidney injury among critically ill patients with sepsis: a multicenter, retrospective cohort study	**Follow-up <3mo**
Siham et al.	2024	Characteristics and prognosis of acute renal failure on dialysis in ANCA vasculitis	**PDF not found**
Kumar et al.	2024	Clinical aspects and prognosis evaluation of cirrhotic patients hospitalized with acute kidney injury	**PDF not found**
Zhao et al.	2024	Clinical Characteristics Analysis of EBV-associated Hemophagocytic Lymphohistiocytosis Patients with Acute Kidney Injury	**PDF not found**
Prasad et al.	2024	Community-acquired acute kidney injury in India: data from ISN-acute kidney injury registry	**Follow-up <1y**
Wang et al.	2024	Construction and evaluation of a mortality prediction model for patients with acute kidney injury undergoing continuous renal replacement therapy based on machine learning algorithms	**Follow-up <3mo**

Li et al.	2024	Construction of a predictive model of death for sepsis-associated acute kidney injury	**PDF not found**
Peng et al.	2024	Correlation between serum calcium level and prognosis of acute kidney injury patients with hypercalcemia	**PDF not found**
Lee et al.	2024	Deciphering AKI in Burn Patients: Correlations between Clinical Clusters and Biomarkers	**Follow-up <3mo**
Medina-González et al.	2024	Decrease in platelet count in patients with AKI and its association with major adverse kidney events	**Wrong control group**
Yue et al.	2024	Development and validation of a nomogram for predicting 3-month mortality risk in patients with sepsis-associated acute kidney injury	**PDF not found**
Li et al.	2024	Development and validation of a nomogram for predicting in-hospital death in cirrhotic patients with acute kidney injury	**Follow-up <3mo**
Lee et al.	2024	Differences in the incidence, characteristics, and outcomes of patients with acute kidney injury in the medical and surgical intensive care units	**Follow-up <3mo**
Hirano et al.	2024	Effect of rehabilitation on renal outcomes after acute kidney injury associated with cardiovascular disease: a retrospective analysis	**Wrong control group**
Lin et al.	2024	Elevated Activated Partial Thromboplastin Time as a Predictor of 28-Day Mortality in Sepsis-Associated Acute Kidney Injury: A Retrospective Cohort Analysis	**Follow-up <3mo**
Flannery et al.	2024	Endotrophin as a Biomarker for Severe Acute Kidney Injury and Major Adverse Kidney Events	**Follow-up <3mo**
Ehmann et al.	2024	Epidemiology and Clinical Outcomes of Community-Acquired Acute Kidney Injury in the Emergency Department: A Multisite Retrospective Cohort Study	**Follow-up <1y**
Singh et al.	2024	Etiology, Clinical Profile and Outcome in Patients with Fever, Jaundice and Acute Kidney Injury: A Prospective Study	**PDF not found**
Kamaraj et al.	2024	Etiology, risk factors and outcome of acute kidney injury in a medical intensive care unit	**PDF not found**
Ieda et al.	2024	Exploration of Risk Factors of the Onset of Antibiotics-induced Acute Kidney Injury and Its Transfer to Chronic Kidney Disease Using the Medical Information Database	**Not in English**
Korla et al.	2024	Exploring Acute Kidney Injury: A Prospective Analysis of Etiology, Clinical Presentation, and Outcome in a Tertiary Care Hospital Setting	**PDF not found**
Yu et al.	2024	EXPRESS: Association between acetaminophen and risk of mortality in patients with sepsis-associated acute kidney injury: A retrospective cohort study from the MIMIC-IV database	**PDF not found**
Franco Palacios et al.	2024	Factors associated with post-hospitalization dialysis dependence in ECMO patients who required continuous renal replacement therapy	**Wrong control group**
AlSahow et al.	2024	Health Care Access, Socioeconomic Status, and Acute Kidney Injury Outcomes: A Prospective National Study	**Follow-up <3mo**
Collett et al.	2024	IL-17A Levels and Progression of Kidney Disease Following Hospitalization with and without Acute Kidney Injury	**Wrong control group**
Petersen et al.	2024	Impact of Acute Kidney Injury After Transcatheter Aortic Valve Replacement: A Nationwide Study	**Follow-up <1y**
Fujita et al.	2024	Impact of severe acute kidney injury on short-term mortality in urosepsis	**Follow-up <3mo**
Lodise et al.	2024	Incidence of acute kidney injury (AKI) and its impact on patient outcomes among adult hospitalized patients with carbapenem-resistant Gram-negative infections who received targeted treatment with a newer β-lactam or β-lactam/β-lactamase inhibitor-, polymyxin- or aminoglycoside-containing regimen	**Follow-up <3mo**
Said et al.	2024	Incidence, predictors and early outcomes of acute kidney injury among patients undergoing abdominal surgery at Bugando Medical Centre, Mwanza, Tanzania	**PDF not found**
Mena et al.	2024	Incidence, Recognition, and Follow-up of Laboratory Evidence of Acute Kidney Injury in Primary Care Practices: Analysis of 93,259 Creatinine Results	**Wrong control group**
Cheng et al.	2024	Incidence, risk factors and outcome of postoperative acute kidney injury in China	**Follow-up <3mo**
Lin et al..	2024	Incidence, risk factors, and outcomes of acute liver injury in hospitalized adults with acute kidney injury: a large multicenter study	**Follow-up <3mo**
Kim et al.	2024	Influence of contrast medium on long-term renal function and outcomes in patients with septic acute kidney injury: A propensity-matched cohort study	**Wrong control group**
Liu et al.	2024	Investigation of renal perfusion and pathological changes in patients with acute kidney disease and tubulointerstitial nephritis using intravoxel incoherent motion and arterial spin labelling MRI: a prospective, observational study protocol	**Wrong study design**
Murphy et al.	2024	Kidney Outcomes with Sodium-Glucose Cotransporter-2 Inhibitor Initiation after AKI among Veterans with Diabetic Kidney Disease	**Wrong control group**
Gordon et al.	2024	Long-term renal function after burn-related acute kidney injury with continuous renal replacement therapy	**Wrong control group**
Gao et al.	2024	Machine learning-based prediction of in-hospital mortality for critically ill patients with sepsis-associated acute kidney injury	**Follow-up <3mo**
Lee et al.	2024	Mortality associated with the neutrophil-lymphocyte ratio in septic acute kidney injury requiring continuous renal replacement therapy	**Follow-up <3mo**
Lehmann et al.	2024	Multiple blood gas variables predict AKI survival in an independent manner	**Follow-up <3mo**
Chen et al.	2024	Neutrophil Percentage as a Potential Biomarker of Acute Kidney Injury Risk and Short-Term Prognosis in Patients with Acute Myocardial Infarction in the Elderly	**Follow-up <3mo**
Vestergaard et al.	2024	Oral anticoagulant treatment and risk of kidney disease—a nationwide, population-based cohort study	**Wrong control group**
Salahuddin et al.	2024	Outcomes of Acute Kidney Injury in Patients Requiring Dialysis in a Tertiary Care Hospital of a Developing Country	**PDF not found**
Gomes et al.	2024	Phenotypes of Dialysis-Requiring Acute Kidney Injury and Associations with Mortality in a South American Population	**Follow-up <3mo**
Lee et al.	2024	Plasma SCUBE2 as a novel biomarker associates with survival outcomes in patients with sepsis-associated acute kidney injury	**Follow-up <3mo**
Buckenmayer et al.	2024	Pre-existing chronic kidney disease, aetiology of acute kidney injury and infection do not affect renal outcome and mortality	**Wrong control group**
Pan et al.	2024	Recovery Dynamics and Prognosis after Dialysis for Acute Kidney Injury	**Wrong control group**
Enghard et al.	2024	Recovery or progression—AKI-CKD transition	**Not in English**
Ling et al.	2024	Red blood cell distribution width to albumin ratio is linked to all-cause mortality in critically ill patients with acute kidney injury: a retrospective cohort study	**Follow-up <3mo**
Ya-fen et al.	2024	Reduction in NGAL at 48 h predicts the progression to CKD in patients with septic associated AKI: a single-center clinical study	**Wrong control group**
Sejpal et al.	2024	Renal Functional Reserve in Acute Kidney Injury Patients Requiring Dialysis	**Follow-up <3mo**
Rüsing et al.	2024	Renal outcome in multiple myeloma patients with cast nephropathy: a retrospective analysis of potential predictive values on clinical and renal outcome	**Follow-up <1y**
Hou et al.	2024	Risk Factors and Prognosis of Acute Kidney Injury in Hospitalised Sepsis Patients	**Follow-up <3mo**
Fan et al.	2024	Risk factors for progression to chronic kidney disease in patients with cardiac valve replacement surgery-associated acute kidney injury	**PDF not found**
White et al.	2024	Sepsis-associated acute kidney injury in patients with chronic kidney disease: Patient characteristics, prevalence, timing, trajectory, treatment and associated outcomes	**Follow-up <1y**
Erfurt et al.	2024	Serum Nostrin-A risk factor of death, kidney replacement therapy and acute kidney disease in acute kidney injury	**Wrong control group**

Alcantar-Vallin et al.	2024	SGLT2i treatment during AKI and its association with major adverse kidney events	**Wrong control group**
Kallur et al.	2024	The impact of acute kidney injury stages on the outcomes of veno-arterial extracorporeal membrane oxygenation	**Follow-up <3mo**
Wang et al.	2024	The incidence, risk factors, and prognosis of acute kidney injury in patients after cardiac surgery	**Follow-up <3mo**
Jiang et al.	2024	The influence of gender on the epidemiology of and outcome from sepsis associated acute kidney injury in ICU: a retrospective propensity-matched cohort study	**Wrong control group**
Wang et al.	2024	The Relationship Between Acute Kidney Injury in Sepsis Patients and Coagulation Dysfunction and Prognosis	**Follow-up <3mo**
Horie et al.	2024	Trajectory pattern of serially measured acute kidney injury biomarkers in critically ill patients: a prospective observational study	**Follow-up <3mo**
León-Román et al.	2024	Transient acute kidney injury after chimeric antigen receptor T-cell therapy in patients with hematological malignancies	**Follow-up <3mo**
Valdenebro et al.	2024	Transitions and Long-Term Clinical Outcomes in Patients Admitted in Intensive Care Units Receiving Continuous Renal Replacement Therapy	**Follow-up <1y**
Li et al.	2024	Trends and Outcomes in Sepsis Hospitalizations With and Without Acute kidney injury: A Nationwide Inpatient Analysis	**Follow-up <3mo**
Ma et al.	2024	Urinary cytokeratin 20 as a predictor for chronic kidney disease following acute kidney injury	**Follow-up <3mo**

Table S4. Risk of bias assessment

	Selection Total (max 4 points)	Comparability Total (max 2 points)	Outcome Total (max 3 points)	Total	Conclusion
Choi et al. (2010)	3	2	2	7	High
Thakar et al. (2011)	3	1	2	6	Moderate
Wu et al. (2011)	4	1	2	7	High
James et al. (2011)	3	2	2	7	High
Jones et al. (2012)	4	0	2	6	Moderate
Bucaloiu et al. (2012)	4	2	2	8	High
Rydén et al. (2014)	3	2	3	8	High
Chawla et al. (2014)	3	2	2	7	High
Xu et al. (2015)	3	2	2	7	High
Arora et al. (2015)	3	1	2	6	Moderate
Grams et al. (2016)	3	2	2	7	High
Heung et al. (2016)	3	2	2	7	High
Gameiro et al. (2016)	4	2	3	9	High
Grams et al. (2016)	3	2	2	7	High
Helgadottir et al. (2016)	3	2	3	8	High
Chew et al. (2017)	3	2	2	7	High
Andreis et al. (2017)	3	0	2	5	Moderate
Wu et al. (2017)	4	2	2	8	High
Chawla et al. (2017)	3	0	2	5	Moderate
Sawhney et al. (2017)	4	1	2	7	High
Palomba et al. (2017)	4	2	2	8	High
Horne et al. (2017)	4	2	2	8	High
Zhang et al. (2018)	4	1	2	7	High
Pourafkari et al. (2018)	3	2	2	7	High
Helgason et al. (2018)	3	2	3	8	High
Neyra et al. (2018)	4	2	2	8	High
Takahashi et al. (2018)	4	2	3	9	High
Azzalini et al. (2018)	3	0	3	6	Moderate
Yeh et al. (2019)	4	2	2	8	High
Wang et al. (2019)	3	0	2	5	Moderate
Zhang et al. (2019)	4	1	2	7	High
James et al. (2019)	3	2	2	7	High
Chaudhury et al. (2019)	4	2	2	8	High
Jamme et al. (2019)	3	1	2	6	Moderate
Mizota et al. (2019)	4	2	2	8	High
Wang et al. (2020)	3	0	3	6	Moderate
Bhatraju et al. (2020)	3	1	3	7	High
Lysak et al. (2020)	3	1	2	6	Moderate
Foxwell et al. (2020)	4	0	3	7	High
Jiang (2020)	3	2	2	7	High
Hsu et al. (2020)	3	2	3	8	High
Menez et al. (2021)	3	0	3	6	Moderate
Ikizler et al. (2021)	3	1	3	7	High
MacLaughlin et al. (2021)	3	1	3	7	High
Cho et al. "Chronic pr..." (2021)	4	0	3	7	High
Cho et al. "Clinical sign…" (2021)	3	1	2	6	Moderate
Reis et al. (2021)	4	0	2	6	Moderate
See et al. (2021)	4	2	3	9	High
Glasbey (2021)	4	1	3	8	High
Long et al. (2021)	4	0	2	6	Moderate
May et al. (2021)	4	2	2	8	High
Choe et al. (2021)	3	2	2	7	High
Go et al. (2021)	3	1	2	6	Moderate
Carias et al. (2022)	3	0	2	5	Moderate
Lyu et al. (2022)	3	2	2	7	High
Nishio et al. (2022)	4	0	2	6	Moderate
Chang et al. (2022)	3	1	2	6	Moderate
Privratsky et al. (2022)	4	2	2	8	High
Chen et al. (2022)	3	2	2	7	High
Lertussavavivat et al. (2022)	4	0	2	6	Moderate
Horne et al. (2023)	4	2	3	9	High
Li et al. (2023)	3	2	3	8	High
Xu et al. (2023)	4	1	3	8	High
Phannajit et al. (2023)	3	2	2	7	High
Chou et al. (2023)	4	2	2	8	High
Nishimoto et al. (2023)	4	2	2	8	High
Colacchio et al. (2023)	4	0	2	6	Moderate
Peerapornratana et al. (2023)	4	2	2	8	High
Medunjanin et al. (2024)	3	2	2	7	High
Zlatanovic et al. (2024)	3	0	2	5	Moderate
